# Full-Length Isoform Sequencing Reveals Novel Transcripts and Substantial Transcriptional Overlaps in a Herpesvirus

**DOI:** 10.1371/journal.pone.0162868

**Published:** 2016-09-29

**Authors:** Dóra Tombácz, Zsolt Csabai, Péter Oláh, Zsolt Balázs, István Likó, Laura Zsigmond, Donald Sharon, Michael Snyder, Zsolt Boldogkői

**Affiliations:** 1 Department of Medical Biology, Faculty of Medicine, University of Szeged, Szeged, Hungary; 2 Department of Genetics, School of Medicine, Stanford University, Stanford, California, United States of America; 3 Institute of Plant Biology, Biological Research Centre, Hungarian Academy of Sciences, Szeged, Hungary; Queen's University, CANADA

## Abstract

Whole transcriptome studies have become essential for understanding the complexity of genetic regulation. However, the conventionally applied short-read sequencing platforms cannot be used to reliably distinguish between many transcript isoforms. The Pacific Biosciences (PacBio) RS II platform is capable of reading long nucleic acid stretches in a single sequencing run. The pseudorabies virus (PRV) is an excellent system to study herpesvirus gene expression and potential interactions between the transcriptional units. In this work, non-amplified and amplified isoform sequencing protocols were used to characterize the poly(A^+^) fraction of the lytic transcriptome of PRV, with the aim of a complete transcriptional annotation of the viral genes. The analyses revealed a previously unrecognized complexity of the PRV transcriptome including the discovery of novel protein-coding and non-coding genes, novel mono- and polycistronic transcription units, as well as extensive transcriptional overlaps between neighboring and distal genes. This study identified non-coding transcripts overlapping all three replication origins of the PRV, which might play a role in the control of DNA synthesis. We additionally established the relative expression levels of gene products. Our investigations revealed that the whole PRV genome is utilized for transcription, including both DNA strands in all coding and intergenic regions. The genome-wide occurrence of transcript overlaps suggests a crosstalk between genes through a network formed by interacting transcriptional machineries with a potential function in the control of gene expression.

## Introduction

**Fig 1:**
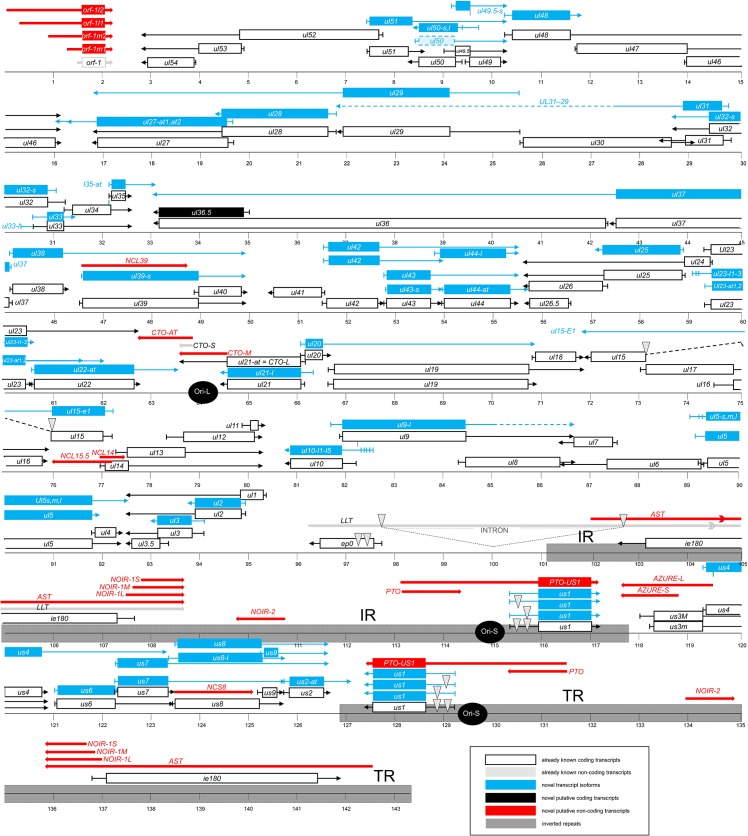
The transcriptome of the pseudorabies virus. The updated version of the PRV genome is composed of 67 protein-coding genes and 20 putative non-coding genes. The coding transcripts described earlier are depicted as white rectangles with black frames. In polycistronic transcripts, only the most upstream ORFs are illustrated by rectangles, while the downstream genes are represented by arrow lines. The already identified non-coding RNAs are represented as dark-gray arrow-rectangles. The novel putative protein-coding gene (*ul36*.*5*) is illustrated as a black rectangle and the novel putative non-coding genes are depicted as red arrow lines. The novel mono- and polycistronic transcripts are indicated with blue rectangles and arrow lines. The light-blue rectangle with dashed border lines indicates a transcript containing antisense sequences of the *ul50* gene at its most upstream region. The long dark-gray rectangles represent the two IR regions of the viral genome; black circles indicate the three replication origins. Abbreviations within the name of the transcripts: ‘s’: small TSS variant; ‘m’: medium-size TSS isoform; ‘l’: long TSS variant; ‘at’: alternative TES variant.

The pseudorabies virus (PRV), an alphaherpesvirus with a broad host range, causes fatal encephalitis in a wide variety of animals, with the exception of its natural reservoir, the adult pig. It is a commonly employed model organism in studies of the molecular pathogenesis of herpesviruses [1,2], for labeling neural circuits [3–5] and for the delivery of genetically-encoded fluorescent activity markers to the neurons [6]. The genomes of viruses are very compact, composed mainly of protein-coding genes and short intergenic regions. The PRV genome contains a unique long (UL) and unique short (US) region, the latter bracketed by inverted repeat (IR) sequences. PRV DNA (upgraded with our own data) contains 67 protein-coding and 20 RNA genes (KJ717942.1) (Fig 1). Similarly as for other herpesviruses, most of the PRV genes are organized into polycistronic transcriptional units, which are typical in prokaryotes, but rare in higher-order organisms [7–9].

Herpesvirus genes are expressed in a temporally ordered cascade and grouped into three kinetic classes. The protein products of immediate-early (IE) genes are required for the transcription of both early (E) genes encoding the synthetic machinery of DNA, and late (L) genes specifying the structural elements of the virus. L genes can be subdivided into leaky late (L1 or E/L) and true late (L2 or L) classes depending upon whether DNA replication is an absolute prerequisite for their expression (this is the case for L2 genes). While the herpes simplex virus (HSV) expresses 5 IE genes, the PRV genome contains only a single one, the immediate-early 180 (*ie180*) gene, which encodes an essential transcriptional transactivator [10]. Kinetic analysis of the PRV transcriptome faces a serious problem due to the polycistronic organization of the viral genes. Previous approaches to analysis of the herpesvirus transcriptome have used microarrays [11], Illumina sequencing, and real-time reverse transcription PCR (RT^2^-PCR) analysis [12]. However, the identification of transcript isoforms, including splice and length variants, with these techniques is difficult or impossible.

Alternative splicing expands the information content of genomes by producing multiple messages from a single gene. Investigations aimed at cracking the ‘splicing code’ have concluded that it is determined by multiple interactions between *cis*- and *trans*-acting factors, but the precise mechanisms are not well understood [13]. The spliced isoforms can have similar or antagonistic functions [14]. Apart from our recent report on an early protein 0 (EP0) transcript [15], the following spliced PRV RNAs have so far been described: US1[16,17], UL15 [18]) and LLT [19].

Despite the fact that hardly more than one per cent of the mammalian genome encodes protein sequences, a large proportion of the DNA is transcriptionally active, producing non-coding RNA molecules (ncRNAs; [20]). Debate is ongoing as to whether this pervasive transcription represents mere transcriptional noise for the most part, or whether these transcripts have still unidentified functions [21]. The most abundant and least annotated class of ncRNAs is the long ncRNAs (lncRNAs [22]), which are defined as transcripts exceeding 200 nucleotides. A large proportion of murine and human DNA was recently reported to encode a wide variety of lnc-RNAs [23]. Many protein-coding genes specify lncRNAs transcribed from the plus strand as templates, which are called antisense lncRNAs. The latency-associated transcript (LAT) of HSV was described as the first lncRNA of viruses [24]. A spliced 8.4-kb antisense RNA, termed long latency transcript (LLT), is synthesized from the complementary DNA strand of *ie180* and *ep0* genes and is controlled by the LAT promoter of PRV [10].

The original genome sequence of the PRV was a composite generated from six viral strains [25] and determined using the traditional Sanger method. The complete genome of PRV strain Ka and other strains have been sequenced both by Sanger capillary sequencing [26] and by Illumina deep sequencing [27–29]. We earlier used the Pacific Biosciences (PacBio) platform to sequence the wild-type Kaplan (Ka) strain of the PRV genome [30].

In this report, PacBio RS II DNA sequencing technology was used to characterize the overall transcriptome of PRV strain Ka. This technique is based on single-molecule sequencing chemistry with real-time detection (SMRT), which allows the sequencing of long stretches of genomic DNAs or transcripts without PCR amplification or fragmentation. We also employed an amplification-based isoform sequencing (Iso-Seq) protocol. Long-read sequencing allows the straightforward identification of alternatively transcribed or processed transcripts, polycistronic transcription units, and other long DNA or cDNA sequences. Moreover, the PacBio platform is especially suitable for the sequencing of small genomes with high GC contents and large amounts of repetitive sequences [30,31]. The PRV genome has a very high overall GC content (~74%) and many repetitive elements, and is therefore difficult to sequence with either the Sanger or the second-generation short-read techniques. PacBio sequencing has the important advantage over other methods in that it does not produce systematic errors and any that arise are therefore easily corrected thanks to its high consensus accuracy [32]. In this study, we wished to reevaluate the currently available knowledge concerning the structures of PRV transcripts by using polyadenylation-sequencing (PA-Seq) based on the PacBio RS II platform, which can identify all poly(A)^+^ RNA molecules generated in cultured porcine kidney (PK-15) cells productively infected with the virus.

## Materials and Methods

### Cells and viruses

Porcine kidney [PK-15; American Type Culture Collection (ATCC)] epithelial cells were used for the propagation of pseudorabies virus strain Kaplan (PRV-Ka). PK-15 cells were cultivated in Dulbecco’s modified Eagle medium supplemented with 5% fetal bovine serum (Gibco Invitrogen) and 80 *μ*g gentamycin/ml at 37°C and 5% CO_2_. For the preparation of virus stocks, rapidly-growing semi-confluent PK-15 cells were infected at a multiplicity of infection (MOI) of 10 plaque-forming unit (pfu)/cell, followed by incubation at 37°C and 5% CO_2_ until a complete cytopathic effect was observed. The infected cells were then frozen and thawed three times, followed by centrifugation at 10,000 g for 15 min. The titer of the virus stock was determined in PK-15 cells. Six parallel infections were carried out for each time point. For all experiments, cells were infected with a high MOI (10 pfu/cell) and incubated for 1 h, followed by removal of the virus suspension and washing of the cells with phosphate-buffered saline (PBS). After the addition of new medium to the cells, they were incubated for 1, 2, 4, 6, 8 or 12 h for the non-amplified technique and for 1, 4 or 8 h for the amplified protocol, and both were mixed for sequencing. Each experiment was carried out in duplicate. Mock-infected cells were used as controls, which were otherwise treated in the same way as the infected cells. We used the following mutant viruses for the analysis of flip-flop mechanism: *ep0*-KO (the early protein 0 gene was deleted[33]), *vhs*-KO (the virion host shut-off gene was deleted [34]), *us1*-KO (*us1* gene deletion; [35]), and *ul54*-KO (*ul54* gene deletion, unpublished).

### PacBio RS II sequencing

Polyadenylated RNAs were isolated from the total RNA by using the Oligotex mRNA Mini Kit (Qiagen) according to the kit instructions for the Oligotex mRNA Spin-Column Protocol.

#### Non-amplified Iso-Seq protocol

The Poly(A^+^) fractions of total RNAs were quantified through use of the Qubit RNA HS Assay Kit (Life Technologies), followed by conversion to cDNAs with the SuperScript Double-Stranded cDNA Synthesis Kit (Life Technologies; the included first strand enzyme was changed to SuperScript III Reverse Transcriptase). The reverse transcription (RT) reactions were primed with Anchored Oligo(dT)_20_ primers (Life Technologies). The cDNAs obtained were quantified with the Qubit HS dsDNA Assay Kit (Life Technologies).

SMRTbell sequencing libraries were generated by using the PacBio DNA Template Prep Kit 2.0 and the Pacific Biosciences template preparation and sequencing protocol for Very Low (10 ng) Input 2 kb libraries with carrier DNA (pBR322, Thermo Scientific). SMRTbell templates were bound to polymerases by using the DNA polymerase binding kit XL 1.0 (part #100-150-800) and v2 primers. The polymerase-template complexes were bound to magbeads with the Pacific Biosciences MagBead Binding Kit. The SMRTBell libraries were analyzed for length and concentration through use of the Agilent 2100 Bioanalyzer. DNA sequencing was carried out with a Pacific Biosciences RS II sequencer using P5-C3 chemistry. Movie lengths were 180 min.

#### Amplified Iso-Seq protocol

Poly(A)^+^ RNAs were purified from total RNA samples by using the Oligotex mRNA Mini Kit (Qiagen), and were converted to cDNAs. The cDNA production and the SMRTbell library preparation were carried out via the protocol described by PacBio: Isoform Sequencing (Iso-Seq) using the Clontech SMARTer PCR cDNA Synthesis Kit and No Size Selection (for the analysis of short transcripts) or Manual Agarose-gel Size Selection (analysis of long transcripts). Briefly, the first-strand cDNAs were generated by using the SMARTer PCR cDNA Synthesis Kit (Clontech).

No size selection: Single-stranded cDNAs (sscDNAs) were amplified by PCR (16 cycles, based on the Test Amplification), using the KAPA HiFi Enzyme (Kapa Biosystems). 500 ng of each cDNA sample was used for the SMRTbell template preparation, using the PacBio DNA Template Prep Kit 2.0. Manual Agarose-gel Size Selection: KAPA HiFi Enzyme was used for the PCR reactions. Two different PCR reactions were used to obtain different transcripts. Twelve PCR cycles and 1:45 min extension were set for the amplification of transcripts between 2–3 kb. Fifteen cycles and 3 min extension was used for the longer transcripts.

The random primer-based PacBio sequencing was carried out exactly as above, except that instead of the oligodT primer, adapter-linked GC-rich random primers were used for the RT reaction.

SMRTbell sequecing templates were bound to polymerases by using the DNA/Polymerase Binding Kit P6 (P/N 100-356-300) and v2 primers. The polymerase-template complexes were bound to magbeads with the PacBio MagBead Binding Kit. The samples were analyzed on the Agilent 2100 bioanalyzer. Sequencing reactions were performed by using the PacBio RS II sequencer with DNA Sequencing Reagent 4.0 (P/N 100-356-200). Movie lengths were 240 min (one movie was recorded for each SMRT cell). The PacBio Iso-Seq protocol (SMRT Analysis version v2.3.0.[36].) was used for transcriptome data analysis.

### Data analysis and visualization

The Bio.motifs package of Biopython [37] was used for identification of the potential polymerase II binding sites and polyA signals. The polymerase II binding motifs were obtained from the JASPAR PolII database [38]. The JASPAR count matrices were converted to the position-weight-matrices and position-specific scoring matrices (PSSM) using the PRV-specific background. The PSSMs were used for a motif search. Score thresholds were generated for the PRV background sequence. Generation of the PSSM for the polyA signal was based on literature data [39]. The PolyApred support vector machine-based method was also used for the prediction of polyadenylation signals [40].

### Illumina sequencing

For Illumina sequencing, RNAs were isolated from cells in various stages of infection up to 24 h pi, and afterwards mixed for library preparation, in order to obtain a wide spectrum of PRV transcripts (mixed infection kinetics). Strand-specific total RNA libraries were prepared for paired-end 100 nt sequencing by using the Illumina-compatible ScriptSeq v2 RNA-Seq Library Preparation Kit (Epicentre). For PA-Seq, a single-end library was constructed through the use of custom anchored adaptor-primer oligonucleotides with an oligo(VN)T20 primer sequence. Anchored primers compensate for the loss in throughput due to the high fraction of reads containing solely adenine bases on the use of conventional oligo(dT) primers. Transcriptome sequencing was performed on an Illumina HiScanSQ platform. FastQC v0.10.1 was used to check the quality of raw read files. Reads were aligned to the pig genome (assembly: Sscrofa10.2) and subsequently to the PRV KJ717942.1 reference genome, using Tophat v2.09 [41]. The ambiguous reads were eliminated from further analysis. The mapping for PA-Seq analysis was performed by using the Bowtie2 program [42], followed by peak detection with HOMER in strand-specific mode, with adjustments for the peak qualities of oligo(dT) primed libraries. We used in-house scripts for the assignment of peak categories based on the following criteria for the abundant transcripts: the presence of a polyadenylation signal (PAS) in the 50-nt region upstream from the poly(A) site and the presence of at least 2 consecutive adenine mismatches in at least 10 independent reads at the poly(A) site. Annotation and visualization were carried out in the Artemis Genome Browser v15.0.0 [43]and IGV v2.2 [44].

### PCR

PCR analysis was used for the validation of putative novel non-coding transcripts of PRV. Total RNA samples were converted to single-stranded cDNAs by using SuperScript III reverse transcriptase (Life Technologies) according to the manufacturer’s instructions. The cDNAs were amplified with the Veriti Thermal Cycler (Applied Biosystems), using AccuPrime^™^ GC-Rich DNA Polymerase (Invitrogen). The running conditions were as follows: 3 min at 95°C, followed by 30 cycles of 92°C for 30 s (denaturation), 60°C for 30 s (annealing), and 72°C for 10 s (extension). Final elongation of 10 min at 72°C was set. The primers used in this study are listed in S1 Table.

### Real-time RT-PCR

Reverse transcription reactions were carried out by using 70 ng of total RNA as template, Superscript III enzyme (Life Technologies) and anchored oligo(dT) primers.

Real-time PCR reactions were performed in a volume of 20 *μ*l with Absolute QPCR SYBR Green Mix (Thermo Scientific) containing 7 *μ*l of cDNA solution diluted 10-fold, 1.5 *μ*l of forward and 1.5 *μ*l of reverse primers (10 *μ*M each). 28S ribosomal (r)RNA was used as a reference gene in each run. The conditions for the PCR amplification were as follows: 15 min at 95°C for the enzyme activation, followed by 30 cycles of 94°C for 25 s (denaturation), 60°C for 25 s (annealing), and 72°C for 6 s (synthesis). Relative expression ratios (*R*) were calculated via the following formula:

$$\bar{R=\frac{{\left(E_{sample\text{ max}}\right)}^{Ct_{sample\text{ max}}}}{{\left(E_{sample\;}\right)}^{Ct_{sample}}}}:\bar{\frac{{\left(E_{ref\text{ max}}\right)}^{Ct_{ref\text{ max}}}}{{\left(E_{ref}\right)}^{Ct_{ref}}}},$$

where E is the amplification efficiency, Ct is the threshold cycle number, “sample” refers to the examined viral transcript and “ref” is the 28S rRNA (internal control). The cDNAs were normalized to 28S cDNAs by using the Comparative Quantitation module of the Rotor-Gene Q software (Version 2.3.1, Qiagen), which automatically calculates the efficiency of the reaction. Thresholds were also set by the software.

### Northern blot analysis

**Fig 2:**
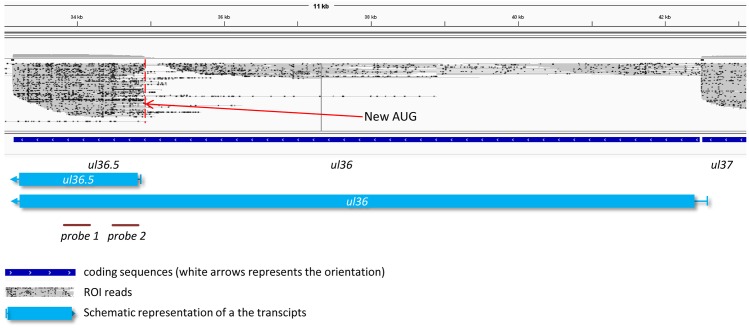
*Ul36*.*5*, a novel gene. Integrative Genome Viewer (IGV) representation of ROIs showing the existence of a novel gene embedded within the ul36 gene. The two probes used for Northern blotting are indicated as solid lines.

Northern blotting was performed as described by Ausubel et al. [45] in the following way: 10 μg RNA from PRV-infected PK-15 cells and 10 μg RNA from non-infected cells were separated on 1% formaldehyde agarose gel. The RNA was blotted by capillary blotting to a positively charged nylon membrane (Hybond-N, Amersham). Two non-overlapping probes—both mapping within the UL36.5 transcript—were used for the hybridization (Fig 2). Probes 1 and 2 were amplified (see primers in S1 Table) then probes were radiolabeled with DecaLabel DNA Labeling Kit (Thermo Scientific) using 2 MBq α-^32^P dCTP. Hybridization was performed at 42°C overnight in 50 ml hybridization solution (0.1% SDS, 5x Denhardt's solution, 30% deionized formamide, 5 mM EDTA, 0.9 M NaCl, 50 mM Na2HPO4, 100 μg/ml fragmented herring sperm DNA, α-^32^P labeled probe). After hybridization, the filters were washed three times at 65°C with the washing solution (0.2xSSC, 0.1% SDS). Scanning and analyzing of the results were done using Typhoon^™^ FLA 9000 imager and ImageQuant 5.0 software.

### Prediction of *cis*-regulatory sequences of the PRV genes

GPMiner[46] server was used for the prediction of TATA box sequences, together with transcription factor binding sites, using standard parameters. The 5'-ends of newly identified transcripts were assigned to the nearest upstream TATA box prediction.

## Results

### Analysis of the PRV lytic transcriptome with PacBio RS II sequencing

We employed two PA-Seq techniques (both based on the PacBio RS II platform) differing in the library preparation, the SMRTbell and the Iso-Seq template preparation protocols, for the investigation of the lytic transcriptome of PRV (Fig 1, S1 Fig). Immortalized PK-15 cells were infected with a high dose of wild-type virus, and the infection was terminated at various time points within a 12-h infection period. The cDNA libraries were prepared from the poly(A^+^) (PA) fraction of total RNAs, using either anchored [in the non-amplified (SMRT) protocol] or modified (in the amplified protocol) oligo-dT primers for the reverse transcription, followed by sequencing. We applied the SMART technique utilizing the switching mechanism at 5' end of RNA template, which provided full-length cDNAs [47]. The short reads [<300 nucleotides (nts) in length] generated by the PacBio sequencer were excluded from further analyses. We obtained a total of 53,320 viral Reads of Insert (ROI) reads [earlier termed circular consensus sequence (CCS) reads]—with an average ROI length of 1287 nts—representing 68,64 million bases with the non-amplified protocol. The number of ROIs mapping to the pig transcriptome was 93,277. The PRV ROIs (and also the subreads) were mapped to the reference genome (KJ717942.1 [30]) using BLASR (part of the PacBio SMRT Analysis software suite as described at [48]) and GMAP[49] alignment tools. We used all ROIs which were at least 300 base pairs (bp) long in order to exploit all of the available data for our analyses. The amplified protocol yielded altogether ~208,000 ROIs, which were mapped to the PRV genome through the use of BLASR software, resulting in ~ 90,700 mapped reads (representing ~ 155 million bases). The mean length of the mapped ROIs was 1709 nts. Besides the poly(A) tails, sequencing reads were produced from A-rich regions of the transcripts due to the use of PA-Seq. This non-specific process occurred very rarely and never produced more than a unique transcript read, which was excluded from further analysis. A minor fraction of the ROIs possessed both poly(A) and poly(T) tails on both termini of the sequencing reads. This phenomenon was earlier described in a human transcriptome sequenced using the PacBio platform [50]. Over 80% of the poly(A/T)^+^ reads were obtained in the UL10 transcript. The ratio of double tailed UL10 reads to the normal reads was approximately 5%. Dual tailing may be an artifact of PCR; these data were discarded from further analysis. In this study, a random primer-based PacBio Iso-Seq method was also conducted. This amplified protocol produced relatively low coverage sequence data in all four independent trials. Despite this fact, we could use these data to identify novel transcripts, including very long RNA molecules, and to confirm the exact 5’-ends of the transcripts. Some data from our Illumina sequencing (PA-Seq and random primer-based RNA-Seq) were also used, which were not included in our earlier publication [15]. We used strand-specific RNA-Seq library preparation for both random primer-based methods (PacBio and Illumina).

### Transcriptional annotation of PRV genes

#### Determination of 5’- and 3’-ends of the PRV transcripts

The full-length PRV transcripts have mainly been predicted *in silico*. Most of the abundant PRV transcripts have been detected by Northern blot hybridization, and close to a third of them have been analyzed by S1 nuclease mapping or primer extension methods (e.g. [51,52]). However, these techniques cannot determine the 5’-ends of the transcripts with base pair precision. Using PacBio analysis, we determined the exact 5’- and 3’-ends of the RNA molecules (S2 and S3 Tables). We found that most of the transcripts were initiated (transcription start site; TSS) at 15 to 31 bp downstream from the TATA box, with a mean of 23.5 bp (median: 23; mode: 22) (the GenBank sequence KJ717942.1 has been upgraded with the new data). We additionally determined the complete nucleotide sequences of the 5’-UTRs of 5 transcripts UL6, UL11, UL23, UL33 and UL36 RNAs, which have not been annotated with any method previously. The amplified Iso-Seq method was found to be superior to the non-amplified protocol in the establishment of accurate 5’-ends and the detection of low-abundance transcripts due to the attachment of an oligonucleotide to the upstream region of the first strand of the cDNAs. This latter technique provided complete nucleotide sequences spanning the entire length of the transcripts from the poly(A)-tail to the 5'-end without any sequence loss. PRV genes were found to be controlled by several TATA box sequence variants (S2 Fig) producing different levels of transcripts (S2B and S2C Fig).

#### Genes controlled by alternative promoters

PacBio sequencing revealed 19 coding and non-coding genes possessing alternative transcription start sites ranging from 2 to 6 TSSs (S4 Table). However, TATA-less transcripts have been reported to be common in eukaryotic organisms [53]. We found that the *ul50* gene contains three active TATA sequences, one located within the *ul49*.*5* gene and the other two within the *ul49* gene. Functional alternative TATA boxes were also identified in the *ul49*.*5*, *ul32*, *ul44*, *ul21*, *ul5* and *us8* protein-coding genes. Both of the previously annotated TATA boxes of the *us3* gene were shown to be active in our experiments.

It emerged that the *orf-1* gene is controlled in a more sophisticated manner than previously believed: besides the *orf-1* gene, four longer transcripts were identified upstream of the known TSS, which were named ORF-1M1 and M2 (M: medium) and ORF-1L1 and L2 (L: long). Among these five transcripts, only the ORF-1 and the ORF-1M are controlled by a TATA box. The presence of an ORF suggests that this gene encodes a protein. However, the distribution of the GC content within the ORF exhibited a pattern intermediate between the intergenic and protein-coding sequences (S3A Fig), which might indicate that *orf-1* is a pseudogene with accumulated mutations destroying the protein-coding function and therefore the GC preference of the third codon positions as well. For an explanation, the distribution of the high GC content within the three reading frames of the PRV protein-coding genes exhibits a special bias: the G+C bases are primarily accumulated at the silent (generally third) codon positions to an extent close to 100% [54]. This phenomenon provides a unique method for prediction of the coding sequences, since the UTRs and the intergenic sequences do not display such a base distribution pattern. However, the close location of AT-rich packaging and cleavage sequencing can distort the codon usage at the upstream region of the *orf-1* gene. Additionally, we showed that only one of the two predicted TATA boxes is active for the *ul37* and *ul42* genes, at least in the PK-15 cells, since we did not obtain any reads from the distant sequences. Our data also revealed that the *ul9* and the *ul41* genes use different TATA elements from those of predicted *in silico*. The *ul10* gene was shown to produce six different isoforms differing in their TSSs.

#### Polymorphism in the 5’-ends of viral transcripts

Our sequencing analysis revealed extensive variation at the TSSs in all but 35 transcripts. It was observed that the transcripts varied in length from 1 to 19 nts at their 5’-ends (mean: 8.2 nts; median: 8; mode: 11) (S5 Table).

#### Transcripts with alternative poly(A) signals

It was earlier demonstrated that the genes in many eukaryotic organisms produce alternative transcription end sites (TESs) [55–57]. We also observed this phenomenon in the PRV RNAs. Two kinds of variation were distinguished: transcripts were produced by using alternative PASs (S2, S3 and S4 Tables) or the same PASs (S5 Table). Alternative PASs can be separated from each other by one or more genes, or they can be located adjacent to each other without intervening coding genes. Such latter PASs were detected in the *ul44* gene with both Illumina and PacBio sequencing, and in the *ul22* and *ul35* genes by only Illumina sequencing. We also detected PAS variants with the method described by Beaudoing and colleagues [39] in 6 additional PRV genes: *ul27*, *ul35*, *ul44*, *ul22 cto-s* and *us2*. PRV transcripts were found to contain several PAS sequence variants (Fig 1), but only the AATAAA sequence is abundant.

#### Polymorphism in the 3’-ends of the transcripts

With the exception of four transcripts (UL17-16, UL16, NCL15.5 and UL48-47-46), we found that all of the abundant RNA molecules containing a single PAS exhibited significant polymorphism in their 3’-ends. The mean variation in the cleavage site position of the coding sequences was 8.8 nts, with a maximum of 28 nts (median: 7 nts; mode: 7 nts) (S5 Table).

#### Novel transcripts

**Fig 3:**
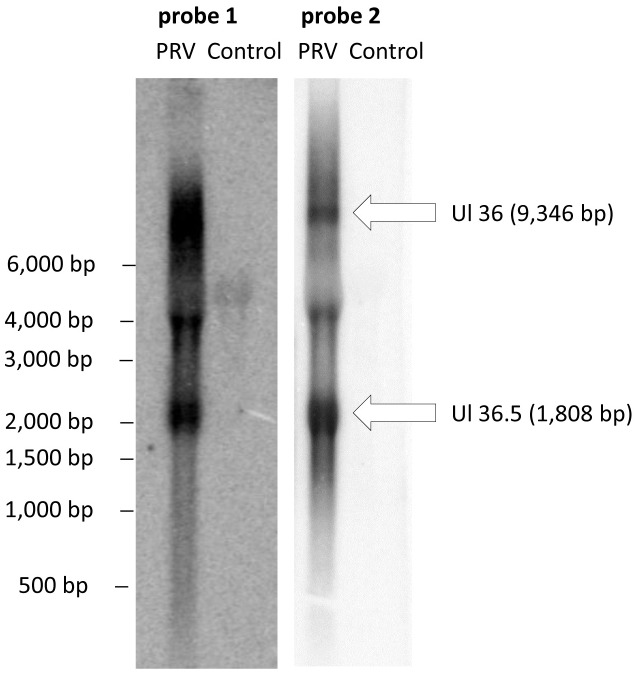
Verification of the existence of ul36.5 transcript by Northern blot analysis. Two non-overlapping radiolabeled DNA fragments both located within the *ul36*.*5* gene were used as probes to detect the RNA molecules produced from this genomic locus. We obtained three bands with both probes: 1. a band corresponding to the 9,436 bp UL36 transcript; 2. a band corresponding to the 1,808 bp UL36.5 transcript and a ~ 4,000 bp band, which might indicate a putative novel transcripts. RNA isolated from PK-15 cells was used as a control.

Our study revealed a novel gene, named *ul36*.*5*, which is embedded within the larger *ul36* gene (Fig 2, S2 Table). The presence of an in-frame ORF within the *ul36* ORF suggests that this gene encodes a shorter version of UL36 protein. This RNA molecule is generated by alternative transcription initiation, resulting in an 1808 bp-long nested transcript located within the interval 34,934–33,127 nts on the KJ717942.1 genome, and sharing 3’-terminals with the UL36 transcript. The *ul36*.*5* gene might produce a truncated version of the UL36 tegument protein, and is composed of 467 amino acids. A putative TATA box for the *ul36*.*5* gene is predicted in the interval 34,959–34,964 nts on the PRV genome. We detected a set of nearly uniform ROI coverage along the length of the gene in every sequencing run, indicating that its expression is regulated independently of the *ul36* gene. We found that UL36.5 was an abundant transcript, while the *ul36* gene was expressed at a low level. The expression of UL36.5 transcript was verified by Northern blot analysis (Fig 3). A low-abundance 4 kbp band has also been detected by using two non-overlapping probes, which might indicate the presence of a middle-sized truncated UL36 transcript. However, we have not obtained PacBio reads of this size therefore further investigations are needed to confirm the existence of the putative RNA molecule.

In this report we have identified 19 novel putative ncRNAs (Fig 1, S2 and S6 Tables).

The *ul15* gene is composed of two exons and an intron; the latter includes the *ul16* and *ul17* genes, both oriented oppositely to the *ul15* gene. The first exon of the *ul15* gene (*ul15-e1*) was also demonstrated to be transcribed separately from the second exon, resulting in a poly(A)-transcript, which we have named NCL15.5 (non-coding UL15.5). This transcript is generated by alternative transcription termination and is 11 nts longer than *ul15-e1*. We identified a weak PAS (AATACA) between 76,173 and 76,168 nts of the PRV genome. The 3’-ends of the NCL15.5 and UL16 transcripts are located immediately adjacent to each other, without an intergenic region between them. Although *ncl15*.*5* encodes the entire amino acid sequence of *ul15-e1*, no stop codon is available in the coding frame. Hence, protein molecules could be produced only by special mechanisms, based, for example, on the use of a distal stop codon in another reading frame through a process such as ribosomal frameshifting, as in the *gag* and *pol* genes of HIV [58]. The amount of NCL15.5 RNA is low relative to that of spliced UL15 RNA (~3.5%).

We recently reported the discovery and characterization of an overlapping ncRNA pair, CTO-S and CTO-L, sharing a common PAS [59]. We identified an additional 3’-end coterminal transcript, termed CTO-M (close to the *o**ri*L–medium size). The transcription of this 960 nt-long ncRNA is predicted to be initiated from a sequence overlapping the PAS of the *ul21* gene. It was demonstrated earlier that PASs can also function as TATA boxes in herpesviruses[60]. No ORF was detected within this RNA gene. The GC distribution of *cto-m* shows no GC-preference in any reading frames (S3B Fig), which suggests that this is a non-coding gene.

We also identified a longer variant of CTO-S, termed CTO-AT (AT: alternative transcription termination; 787 bps), overlapping the UL22-AT, which is an isoform of the UL22 transcript with longer 3’-UTR.

We identified three 3’-coterminal non-coding polyadenylated transcripts, named NOIR1-S, NOIR1-M and NOIR1-L (non-coding RNA in the inverted repeat; short, medium or long variant), located in the IR region of PRV, with sizes of 1293 nts, 1425 nts and 1485 nts, respectively. The *noir1* genes are arranged in different orientations compared to the *ie180* gene. These transcripts are more abundant than the IE180 mRNA. The common PAS of the *noir1* genes is localized in the DNA segment 109278–109283 nts, but we could not identify TATA boxes upstream of these genes, except for the shortest transcript. We also detected longer overlapping 3’ co-terminal transcripts, which may be incomplete reads from the LLT driven by the LAT promoter.

We also detected a 902 nt-long non-coding transcript (termed NOIR2) located in the IR region. The *noir2* gene is situated at a distance of 2721 bp downstream of the *ie180* gene and arranged in a parallel orientation with this transactivator gene, and in a convergent orientation with the *noir1* genes. We could not identify either a putative TATA box or typical PAS sequence (only atypical: AATAGA). We obtained relatively few reads with both PacBio and Illumina platforms at each time point from this RNA as compared with an average viral transcript. The lack of ORFs indicates that all four *noir* genes are lncRNAs. No GC-preference is observed at any reading frames of the *noir* genes (S3C Fig), which suggests *that they are* non-coding genes.

Two overlapping ncRNA genes were also identified near the *ori*S sequences, which were named PTO (proximal to the *o**ri*S; 1098 bps) and PTO-US1 (4087 bps). No complete ROIs were produced from this latter transcript; we can only assume that it is initiated from the nearby *pto* promoter. This transcript overlaps with the *ori*S of the PRV. The PTO-US1 can also be considered to be a US1 transcript with a very long alternative 5’-UTR. No GC-preference is observed at any reading frames (S3D Fig), which suggests that *pto* is a non-coding gene.

An additional pair of coterminal ncRNAs encompassing the US-IR boundary region was identified, and termed AZURE (antisense transcripts in the IR-US overlapping region). The *azure-s* gene codes for an 1198 nt-long lncRNA, which partially overlaps the *us3* gene and a 294-nt segment of the IR region; *azure-l* encodes a 2039 nt-long transcript overlapping partially the *us4* gene and fully the *us3* gene. The PA signal of the AZURE transcripts was located between 117,737 and 117,742 nts, but no TATA boxes were detected for the gene encoding this RNA molecule. There is no GC-preference in those sequences of the *azure* gene (S3D Fig), which do not overlap with the *us3* gene, which suggests that *azure* is a non-coding gene.

We could detect existence of the antisense transcript (AST) controlled by the antisense promoter (ASP), which was predicted by Cheung [19]. The AST and LLT are coterminal with the NOIR-1 transcripts.

#### Truncated transcripts

We detected three polyadenylated transcripts, termed NCL14 (433 bp), NCL39 (2234 bp) and NCS8 (NCS: non-coding US; 1534 bp), whose expressions were prematurely terminated, and therefore lacked their stop codons. The potential function of these truncated RNA molecules, if any, remains to be ascertained.

**Fig 4:**
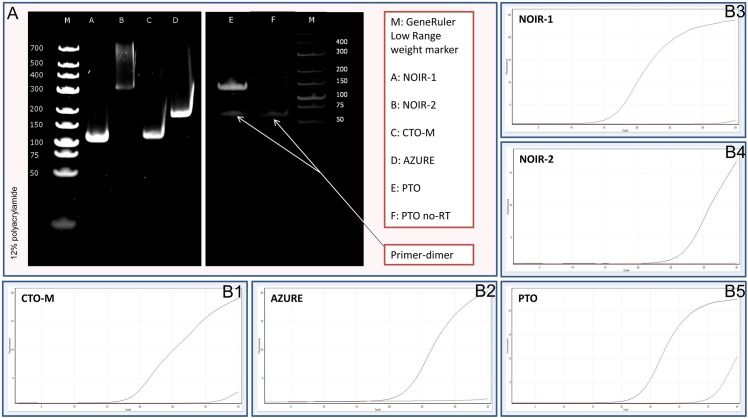
Detection of novel ncRNAs by PCR analysis. Our PacBio analyses identified 19 ncRNAs. We could confirm the expression with traditional (A) and quantitative (B) PCR analyses of those transcripts which were not embedded into a larger transcript. The following novel non-coding transcripts were detected by PCR: (B1) CTO-M (113 bp), (B2) AZURE (189 bp), (B3) NOIR-1 (116 bp), (B4) NOIR-2 (332 bp), and (B5) PTO (143 bp). We included the PTO—no RT control to demonstrate that the low molecular weight fragment is a non-specific primer dimer (indicated by arrows). The amplification curves obtained by RT^2^-PCR are also indicated to demonstrate the specificity of the amplification. The curves of specific transcripts cross the threshold line within 15–25 cycles, while the curves of no-RT controls remain flat.

The expression of the non-parallel overlapping ncRNAs was verified by traditional and quantitative PCR analyses (Fig 4); and the remainder of the transcripts was confirmed by Illumina sequencing and/or random-primer based Iso-Seq sequencing (data not shown). We did not detect the UL8.5, the LAT transcripts and the spliced version of LLT [61] with any of the sequencing techniques used in this study.

#### Novel transcript isoforms

It is currently thought that the expression of the herpesvirus genes follows the general scheme that most of the downstream genes in a tandem gene cluster can be transcribed either as monocistronic mRNAs or as downstream genes in polycistronic (bi-, tri- or tetracistronic) transcripts, while the upstream genes are expressed only as parts of polycistronic RNA molecules. However, our investigations revealed that many upstream genes of 3’-coterminal gene clusters are also expressed as monocistronic transcripts, and some genes believed to have only their own transcriptional termination also share common 3’-ends with other tandem genes (Fig 1, S3 Table). We found that, in addition to polycistronic expression, nine genes were also transcribed individually. Moreover, we identified several previously unrecognized tandem polycistronic units: six bicistronic, seven tricistronic and one tetracistronic transcripts, for which we could determine the 5’-ends (Fig 1, S2 and S7 Tables). Earlier annotations suggest that the *ul20* gene is expressed exclusively as a monocistronic RNA, whereas we found that the UL20-19 bicistronic RNAs are represented in a much higher proportion than the UL20 transcript in infected PK-15 cells. Our study revealed altogether 30 genes that produce novel transcription variants by utilizing one proximate and one or more distant additional PA signals (Fig 1, S2 Table: the longer monocistronic transcript isoforms are labeled with “-AT” affix). The newly discovered mono- and polycistronic transcripts are typically expressed at low levels, which explain why they went undetected previously. The question arises as to whether the only function of these transcripts is to contribute to the proteome of the infected cells, or if they also play other additional roles.

#### Complex transcripts

Complex transcripts contain genes situated in opposite orientations relative to each other (Fig 1, S1 Fig, Table 1). We identified two full-length complex transcripts: UL51-50-49.5–49 and UL50-49.5–49 (Table 1a). The latter transcript is initiated from the sequence overlapping the PAS of the *ul51* gene. Our investigations revealed a widespread expression of very long polycistronic RNAs belonging to this category, whose upstream sequences could not be determined with the PA-Seq techniques. We illustrated these low-abundance RNA molecules as if they were controlled by the promoters of the closest upstream genes oriented in the right direction and gave *ad hoc* names accordingly. However, it is possible that they are shorter and initiated by still unidentified promoters, or even longer driven by more distant promoters. We identified 15 transcripts with incomplete 5’-ends (Table 1b). Five complex transcripts (UL18-15E2-17-16, AZURE-US1-PTO-NOIR2, US2-US1-PTO-NOIR2, NOIR1-NOIR2-PTO-US1and UL31-30-29) were detected only by strand-specific random primer-based PacBio sequencing. With the exception of UL31-30-29, we could not identify their TSSs (their TESs were also undetected; Table 1c). The presence of the antisense RNA sequences on these transcripts allows the confirmation of their existence by RT^2^-PCR analysis and Illumina sequencing, which were carried out in each case (Table 1) We also analyzed those parts of the genome for antisense RNA expression which did not produce PacBio reads at all by RT^2^-PCR and Illumina sequencing, and found extensive transcription from the complementary regions of practically every protein-coding and non-coding gene (Table 1d). The ratio of sense and antisense transcripts was calculated (S8 Table). We must assume that the entire PRV genome produces very long low-abundance complex transcripts.

**Table 1:** Complex transcripts.

**A**	**Full-length complex transcripts**
	UL51-50-49.5–49
	UL50-49.5–49
**B**	**Putative complex transcripts with detected PA**
	ORF-1-UL54-53-52-51
	Ul27-46-47-48-49-49.5–50
	UL30-31-32-33-34-35
	UL36.5-35-34-33-32-31
	UL41-40-39-38-37
	UL35-36.5-36-37-38
	UL40-41-42
	UL26.5-44-43-42-41
	UL44-26.5-26-25-24-23
	UL15-16-17-18-19-20-21
	UL17-16-15-14-13-12-11
	UL11-10-9-8
	UL7-8-9-10
	EP0-LLT-UL1-2-3-3.5
	IE180-LLT-EP0
**C**	**Putative complex transcripts with non-detected PA**
	UL18-15E2-17-16
	UL31-30-29
	AZURE-US1-PTO-NOIR2
	US2-US1-PTO-NOIR2
	NOIR1-NOIR2-PTO-US1
**D**	**Putative complex transcripts with no PacBio detection**
	UL3.5-4-5-6
	UL10-11-12-13-14-15E2-16-17-15E1
	UL22-CTO-UL21-20
	UL21-CTO-UL22-23-24-25-26
	UL46-27-28-29-30

The complex transcripts harbor genes with at least two different orientations. The presence of antisense sequences allows the detection of these transcripts by RT^2-^PCR and DNA sequencing, using strand-specific primers for the reverse transcription. We gave ad hoc names to these transcripts because their missing sequences were only predicted. We also analyzed those parts of the genome for antisense RNA expression which were undetected as parts of complex transcripts. The complex transcripts are categorized in terms of their identified region as follows: full-length complex transcripts (a); putative complex transcripts with identified PA sequences (b); putative complex transcripts with non-identified PA sequences (c); putative complex transcripts not detected with PacBio sequencing at all (d). The ratios of antisense to mRNAs obtained with RT^2^-PCR and Illumina sequencing were calculated. MOI stands for ‘multiplicity of infection’ of PRV used for the infection experiment (high MOI: 10 pfu; low MOI: 0.1 pfu; pfu: plaque-forming units). The antisense parts of the complex transcripts are underlined in Table 1a–d.

### PRV transcriptome profiling

**Fig 5:**
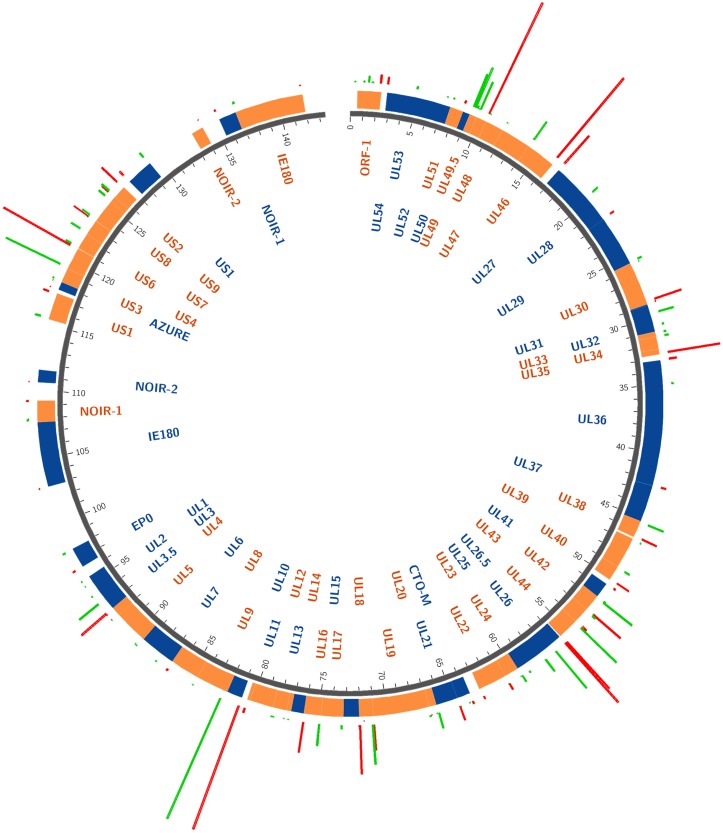
Profiling of the PRV transcriptome. The distribution and amount of the transcription start sites (TSSs) and poly(A) signals (PASs) were represented by circos plots [62]. Genes on the plus strand are drawn in orange and genes on the minus strand are in blue. Green bars show the amount of reads that start at the main TSSs, red bars show the amounts of reads that contain the PASs.

SMRT sequencing was used to evaluate the relative rate of gene expression, which was measured by calculation of the numbers of PAS and TSS sequences, and the ROIs obtained from the gene products (Fig 5) and the relative copy numbers (Table 2) along the entire PRV genome. The *ul10* gene was found to produce the highest amount of transcripts, which was followed by *ul46*, *ul49*.*5*, *us4*, *ul26*.*5*, *ul18*, *ul34* and *ul44*. We earlier revealed by Illumina sequencing that CTO-S was the most abundant PRV transcript, but it is too small (260 bp) for PacBio sequencing and its abundance therefore was underestimated in this study. S4 Fig shows that essentially the entire genome is transcriptionally active.

**Table 2:** Profiling of the PRV transcriptome.

**A**	**TRANSCRIPTS**	**Abundance**	**B**	**3' COTERMINAL TRANSCRIPTS**	**Abundance**
	**ORF-1L2**	22.5 *		**ORF-1L2**	4493.7
	**ORF-1L1**	90.0 *		**ORF-1L1**	
	**ORF-1**	301.4		**ORF-1**	
	**ORF-1M1**	225.0 *		**ORF-1M1**	
	**ORF-1M2**	1050.0 *		**ORF-1M2**	
	**UL54**	948.7		**UL54**	3681.2
	**UL53-54**	1333.6		**UL53-54**	
	**UL52-53-54**	171.2		**UL52-53-54**	
	**UL51**	396.8		**UL51**	396.9
	**UL51-50-49.5–49**	166.3		**UL51-50-49.5–49**	
	**UL50S**	57.6 *		**UL50S**	1318.3
	**UL50**	1272.2		**UL50**	
	**UL50L**	46.1		**UL50L**	
	**UL50-49.5–49**	2.82		**UL50-49.5–49**	
	**UL49.5–49**	18661.2		**UL49.5–49**	65818.1
	**UL49.5-S-49**	22947.1		**UL49.5-S-49**	
	**UL49**	15944.0		**UL49**	
	**UL48**	1.1		**UL48**	55362.1
	**UL48-47-46**	160.1		**UL48-47-46**	
	**UL47-46**	44.5		**UL47-46**	
	**UL46**	11589.6		**UL46**	
	**UL27-AT1**	0.2		**UL27-AT1**	0.2
	**UL27-AT2**	0.2		**UL27-AT2**	0.2
	**UL27**	3641.3		**UL27**	19707.7
	**UL28-27**	24.6		**UL28-27**	
	**UL29-28-27**	24.6 *		**UL29-28-27**	
	**UL28**	**		**UL28**	**
	**UL29**	2275.0		**UL29**	2275.0
	**UL29-L**	8.1 *		**UL29-L**	
	**UL30**	88.3		**UL30**	88.3
	**UL31**	8271.3		**UL31**	15158.7
	**UL32-S-31**	312.0 *		**UL32-S-31**	
	**UL32-31**	1092.1		**UL32-31**	
	**UL33**	1.2		**UL33**	1.2
	**UL33-34-35**	887.7		**UL33-34-35**	28273.1
	**UL33-L-34-35**	111.0 *		**UL33-L-34-35**	
	**UL34-35**	24186.9		**UL34-35**	
	**UL35**	178.9		**UL35**	
	**UL35-AT**	**		**UL35-AT**	**
	**UL36.5**	1085.3		**UL36.5**	4173.9
	**UL36**	34.3 *		**UL36**	
	**UL37-36**	6.9 *		**UL37-36**	
	**UL37**	2819.4		**UL37**	2819.4
	**NCL39**	**		**NCL39**	8594.8
	**UL38**	8594.8		**UL38**	
	**UL38-39-40**	172.5		**UL38-39-40**	7499.1
	**UL39-40**	193.7		**UL39-40**	
	**UL39-S-40**	241.0		**UL39-S-40**	
	**UL40**	2965.0		**UL40**	
	**U41**	1176.9		**U41**	1176.9
	**UL42**	19225.9		**UL42**	19255.9
	**UL42-43**	222.9		**UL42-43**	3044.9
	**UL43**	64.0		**UL43**	
	**UL43S**	2112.8		**UL43S**	
	**UL42-43-44**	219.4		**UL42-43-44**	33744.4
	**UL43-44**	520.2		**UL43-44**	
	**UL44**	26285.8		**UL44**	
	**UL44-L**	170.4		**UL44-L**	
	**UL44-AT**	8.6 *		**UL44-AT**	8.6 *
	**UL26.5**	25624.6		**UL26.5**	42423.5
	**UL26-26.5**	7803.4		**UL26-26.5**	
	**UL25-26-26.5**	1003.3		**UL25-26-26.5**	
	**UL24-25-26-26.5**	72.2		**UL24-25-26-26.5**	
	**UL23-L3**	39.9		**UL23-L3**	3755.5
	**UL23-L2**	219.7		**UL23-L2**	
	**UL23-L1**	1194.1		**UL23-L1**	
	**UL23**	2561.4		**UL23**	
	**UL23-AT**	1.6		**UL23-AT**	1.6
	**UL23-AT2**	0.9		**UL23-AT2**	0.9
	**UL23-22**	107		**UL23-22**	1492.0
	**UL22**	240,8		**UL22**	
	**UL22-AT**	9.2 *		**UL22-AT**	9.2 *
	**CTO-AT**	4.6 *		**CTO-AT**	4.7 *
	**CTO-S**	250.4		**CTO-S**	1384.1
	**CTO-M**	428.2		**CTO-M**	
	**CTO-L**	506.3		**CTO-L**	
	**UL21**	7579.6		**UL21**	7895.4
	**UL21-L**	315.8 *		**UL21-L**	
	**UL20**	1.1		**UL20**	1.1
	**UL20-19**	34.5		**UL20-19**	13793.8
	**UL19**	664.7		**UL19**	
	**UL19-18**	65.7		**UL19-18**	26168.2
	**UL18**	21252.6		**UL18**	
	**UL15**	300.0		**UL15**	706.0
	**UL15E1 -16-17**	129.9		**UL15E1 -16-17**	
	**NCL15.5**	35.9		**NCL15.5**	42.4
	**UL17-16**	1978.7		**UL17-16**	16391.5
	**UL16**	11646.4		**UL16**	
	**NCL14**	17.9		**NCL14**	2644.2
	**UL14-13-12-11**	103.7		**UL14-13-12-11**	
	**UL13-12-11**	6,2		**UL13-12-11**	
	**UL12-11**	459,2		**UL12-11**	
	**UL11**	1377.6 *		**UL11**	
	**UL10**	70112,4		**UL10**	70524.8
	**UL10 -L1**	170 *		**UL10 -L1**	
	**UL10-L2**	76 *		**UL10-L2**	
	**UL10-L3**	38 *		**UL10-L3**	
	**UL10-L4**	95 *		**UL10-L4**	
	**UL10-L5**	38 *		**UL10-L5**	
	**UL9-8-L**	48 *		**UL9-8-L**	849.2
	**UL9-8**	48		**UL9-8**	
	**UL8**	212,2		**UL8**	
	**UL7**	463,1		**UL7**	1094.4
	**UL6-7**	414,9		**UL6-7**	
	**UL5**	7,7		**UL5**	7.7
	**UL5-4**	267.1		**UL5-4**	423.8
	**UL5-4-L**	11.6		**UL5-4-L**	
	**UL5-4-M**	116.1		**UL5-4-M**	
	**UL-5-4-S**	11.6		**UL-5-4-S**	
	**UL4**	14		**UL4**	
	**UL3.5**	13071,6		**UL3.5**	17516.0
	**UL3-3.5**	423,8		**UL3-3.5**	
	**UL3**	**		**UL2-3-3.5**	
	**UL2-3-3.5**	70.7		**UL1-2-3-3.5**	
	**UL2**	**		**UL3**	**
	**UL1-2-3-3.5**	388.4		**UL2**	**
	**Ep0**	1329.5		**Ep0**	1329.5
	**IE180**	542.5		**IE180**	542.5
	**LLT**	2.2 *		**LLT**	4081.9
	**AST**	9.5		**AST**	
	**NOIR1-S**	15.7 *		**NOIR1-S**	
	**NOIR-1M**	857.3		**NOIR1-M**	
	**NOIR-1L**	77.9		**NOIR1-L**	
	**NOIR-2**	20.3 *		**NOIR-2**	20.3*
	**PTO**	7.1 *		**PTO**	7.0 *
	**PTO-US1**	19.0 *		**PTO-US1**	5678.9
	**US1**	5678.9		**US1**	
	**AZURE-L**	9.5		**AZURE**	40.9
	**AZURE-S**	28.4		**AZURE-S**	
	**US3-L-4**	70.1		**US3-L-4**	40863.4
	**US3-4**	615.7		**US3-4**	
	**US4**	32201.2		**US4**	
	**US4-6-7**	3.9		**US6**	626.0
	**US6**	626		**US4-6-7**	9378.5
	**US6-7**	2572.1		**US6-7**	
	**US7**	5594.8		**US7**	
	**US7-8-9**	11.2		**NCS-8**	**
	**US7-8-9-2**	4.6		**US7-8-9**	4482.9
	**NCS-8**	**		**US8-9**	
	**US8-9**	4232.1		**US9**	
	**US8-9-2**	519.9		**US7-8-9-2**	11106.4
	**US9**	202.8		**US8-9-2**	
	**US9-2**	3854.1		**US9-2**	
	**US2**	5046.6		**US2**	
	**US2-AT**	72		**US2-AT**	46.8

The relative amounts of TSS (a) and (b) PAS sequences in the lytic transcriptome were determined in this study by using the non-amplified PacBio data. Values indicated with asterisks are obtained with the amplified method. Transcript isoforms detected by Illumina sequencing are indicated by double-asterisks. ROI read numbers were normalized to the transcript level of pig mitochondria (MT). Normalization was needed because of the parallel samples.

### Splice isoforms

The US1 transcript has been reported to contain two introns [16]. PacBio analysis showed that, besides the published double-spliced transcripts, the other possible combinations of splice isoforms, including the non-spliced and the two single-spliced transcripts, were also expressed (Table 3). PacBio sequencing allowed the precise estimation of the occurrence of splice variants. We also performed such calculations for the UL15 and EP0 transcripts.

**Table 3:** Splice isoforms.

SPLICE ISOFORMS		%
**US1**	**double-spliced**	96.6
	**non-spliced**	1.4
	**single spliced (intron 1)**	1.2
	**single spliced (intron 2)**	0.8
**UL15**	**spliced**	53.3
	**non-spliced**	46.7
**EP0**	**non-spliced**	77.6
	**spliced (intron 1)**	1.7
	**spliced (intron 2)**	20.7

PacBio sequencing revealed that all four splicing combinations of the US1 transcripts are expressed in the lytic cycle of the virus. We calculated the relative proportions of the splice variants in the known spliced transcripts, including US1, EP0 and UL15.

### Transcriptional overlaps between adjacent genes

Our analysis considerably increased the number of already known transcriptional overlaps between neighboring, or distant genes. We showed that practically the entire PRV genome expresses overlapping transcripts.

#### Parallel overlaps

**Fig 6:**
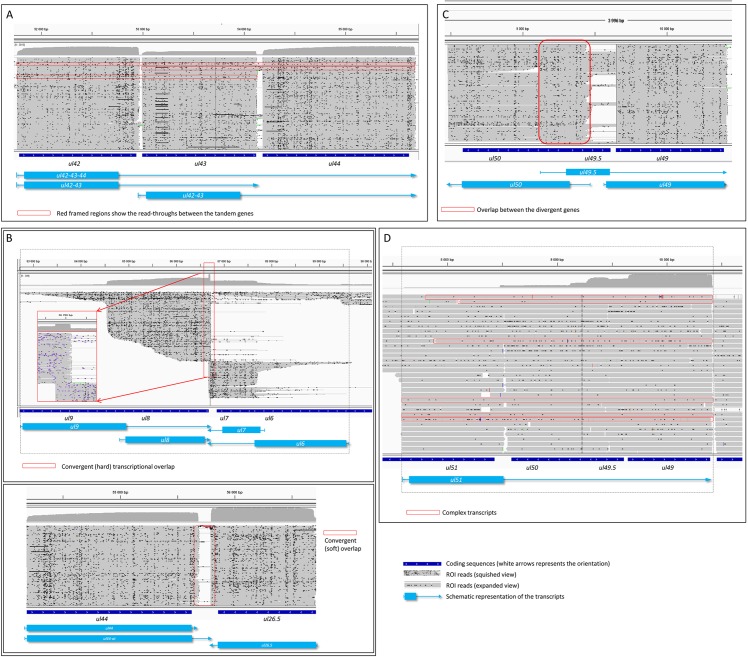
Extensive overlaps between PRV transcripts. Transcriptional overlaps between adjacent (A: parallel; B: convergent “hard” and “soft”; C: divergent) and distal (D) genes. Figures represent the ROIs and the coverage of reads across certain genomic regions. The overlapping transcripts are shown at the bottom of the figures. A, B and C figures illustrate the squished view of the ROIs, while panel D shows the expanded view of the reads.

Either a partial or a full parallel overlap can exist between the tandemly-oriented adjacent PRV genes. Our analysis revealed that most of the tandem genes formed both full and partial overlaps with each other. We detected 18 novel parallel full transcript overlaps, and 20 partial tail-to-head overlap between tandemly oriented genes. This type of overlap is illustrated by using *ul42*, *ul43*, and *ul44* genes as examples (Fig 6a).

#### Convergent overlaps

In other annotations of the PRV genome (NC006151.1 and JF797218.1), only the *ul30* and *ul31* genes form convergent transcriptional overlaps with each other (‘hard’ overlap: all transcripts overlap with each other). We found that the overlap between these two genes is longer than predicted by 204 nts. We also found ‘hard’ tail-to-tail overlaps in the *ul8/7*, *ul50/51* and *us3*/*azure* gene pairs It emerged that the alternative polyadenylation produces convergent overlapping between the *ul44*/*ul26* gene pair, in such a way that the longer UL44 transcript extends to the 3’-UTR region of the Ul26 transcript. The utilization of alternative PASs of the Ul35 and Ul22 transcripts also generates an overlap with the Ul36 and CTO transcripts, respectively. Furthermore, very short intergenic regions were found between the convergently-oriented gene pairs *ul4*/*ul3*.*5* (4 nucleotides between the TESs) and *ul17*/*ul15-e1* (10 nucleotides between the TSSs), which potentially allows interference between the RNA polymerase molecules of the adjacent genes when they are simultaneously transcribed. For example, the *ul9-ul8* and *ul7-ul6* gene pairs form a ‘hard’ overlap, while there is a ‘soft’ overlap between the *ul44* and *ul26*.*5* genes (Fig 6b).

#### Divergent overlaps

Earlier annotations revealed that all 12 divergent partner genes of the PRV form head-to-head overlaps with each other. Two divergent partners display TATA box overlaps, while the other genes overlap in their transcribed regions, including 5’-UTR/5’-UTR, the 5’-UTR/coding region (CR) and CR/CR. PacBio analysis revealed alternative extended 5’-ends of several genes, which provide much longer overlapping regions between the divergent partners. The divergent overlap is illustrated by the *ul50* and *ul49*.*5* gene pairs (Fig 6c).

#### Overlaps between distant genes

An additional form of transcriptional overlaps is produced by non-adjacent genes in a tandem gene cluster, which we term distant parallel overlaps. The existence of transcripts containing genes with various orientations indicates that besides local genes, distal genes can also interact with each other by forming various types of transcriptional overlaps, called complex overlaps. The transcript comprising the *ul51*, *ul50*, *ul49*.*5* and *ul49* genes indicates a complex overlap between distal genes (Fig 6d).

## Discussion

Short-read sequencing has become a widespread approach for the structural and functional annotation of transcriptomes [63–65]. However, this technique is not optimal for the *de novo* characterization of the transcriptome, since it is unable to identify alternatively transcribed and processed transcripts. In this study, a massively parallel sequencing platform based on PacBio’s SMRT sequencing was applied for the analysis of the PRV transcriptome.

We determined the nucleotide sequences of already described RNA molecules with base pair precision. Additionally, we succeeded in identifying a previously transcripts. Our work revealed that most PRV genes are transcribed in various combinations with the genes in their neighborhood, forming so far unidentified mono- and polycistronic transcripts. Most of the novel transcripts are produced at low levels, which hampers their detection by gel-based assays or, because of their overlapping nature, even by PCR. It may be speculated whether the major role of the ORF-containing low-abundance transcripts is to contribute to the viral proteome, or whether they play a role as lncRNAs or precursors for shorter ncRNAs, or perhaps only represent transcriptional noise. Another possibility is that these RNA molecules are mere by-products of a genetic regulatory mechanism based on transcriptional read-through processes [66]. Indeed, transcriptome analysis of antisense lncRNAs and their mRNA partners has revealed the interrelated regulation of expression [67], and in many cases reciprocal expression patterns [68]. Furthermore, the antisense lncRNAs have been shown to block the expression of their mRNA counterparts in cultured human cells [69].

Our PacBio analysis also detected very long polycistronic RNA molecules (called complex transcripts) containing genes in opposite orientations relative to each other. RT^2^-PCR analysis and Illumina sequencing showed that, besides these complex transcripts, almost the entire PRV genome exhibits varying levels of antisense RNA sequences, which are presumably parts of complex transcripts initiated and terminated by sequences of already annotated genes.

In addition to the already known cases, our analysis revealed a wide variety of novel transcriptional overlaps between adjacent and distant genes situated in parallel, convergent or divergent orientations to each other. In our earlier study (Oláh et al., 2015) we could not detect most of the convergent transcriptional overlaps, because the Illumina platform is not ideal for sequencing repetitive sequences, which are common in the intergenic regions, and therefore we did not obtain sequencing reads at these locations. The extensive transcriptional overlaps between the viral genes demand an explanation. One possibility is that this phenomenon is a result of the economic utilization of the viral DNA. However, the full-length overlap of genes does not save any space in the genome, and the total gain with other types of overlaps is minor. We assume that the transcriptional machineries of neighboring or distant genes collide and/or compete with each other[70,71] by forming a meshwork of interactions between the transcriptional machineries, which we called a Transcription Interference Network (TIN), representing a novel layer of genetic regulation [66]. The TIN hypothesis claims that the expression of a gene depends not only on its cis-regulatory sequences, but also on the activity of its proximal and distal neighborhoods. Intriguingly, Rutkowsky and colleagues found that HSV-1 triggered the disruption of transcription termination of cellular, but not viral, genes, which results in extensive transcription of host genes beyond poly(A) sites and into adjacent genes[72]

This study revealed two novel non-coding transcripts (CTO-M and PTO-US1) overlapping the replication origins (*ori*L and *ori*S, respectively) of the virus, and another ncRNA (PTO) which is encoded by a DNA locus situated close to *ori*S. Transcriptions from the genes encoding these RNAs can potentially regulate the onset and the orientation of DNA replication in a manner described earlier for the *cto-s* and *cto-l* genes [59]. The question arises as to whether the *ori*-overlapping transcripts represent a general phenomenon, or their existence is restricted to the PRV. In another study, we detected an overall drop in the transcriptional activity of the genes per viral genome after the onset of DNA synthesis [35]. The existence of *ori*-overlapping transcripts and the repression of global transcription during DNA replication suggest an interaction between the replication and transcription machineries (such as described by Aguilera et al., [73]) along the entire viral genome.

## Conclusions

Our results revealed the feasibility of the deep sequencing of full-length RNA molecules from the transcriptome of a herpesvirus both at a single-molecule level and in amplified samples. Our investigations essentially redefine the transcriptome of the PRV. We demonstrated that herpesviruses exhibit considerably more genetic complexity than predicted from *in silico* ORF-based genome annotations and gel-based assays. Our investigations uncovered that essentially the entire PRV genome is transcriptionally active, including both DNA strands of the coding and intergenic sequences. Identification of a pervasive genome-wide overlapping pattern of PRV transcripts and of *ori*-overlapping RNA molecules raises the possibility for the existence of a genome-wide network exerting joint control on gene expression and replication.

## Supporting Information

S1 Fig Very long novel PRV transcripts with unidentified 5’ or 5’ and 3’-ends. Using PacBio and Illumina RNA sequencing and RT^2^-PCR, we have detected several very long transcripts with unidentified ends. The transcripts with unknown upstream, or upstream and downstream sequences are depicted as thin green rectangles and dashed arrow-lines in their non-sequenced regions. The green rectangles represent the longest sequence obtained by PacBio sequencing. We assume that the long transcripts are controlled by the next available promoter oriented in the appropriate direction. The 3’ transcript ends, if undetected, were predicted to be located at the next available PAS. However, these assumptions remain to be confirmed experimentally. The names that we selected for these transcripts are therefore considered to be ad hoc (the names contain all of the genes located on a transcript, but in this Figure they are abbreviated by using the names of only the first and last genes). The light-blue rectangles with dashed borders are those parts of the transcripts which we had earlier sequenced (but not published) by the Illumina platform, and the dark-gray rectangles inside them are the genomic loci which were analyzed by RT^2^-PCR in this study. The protein-coding transcripts are depicted as white rectangles with black borders, while the non-coding RNAs are illustrated as black arrow lines.
(PDF)

S2 Fig TATA box variants of PRV. (A) Sequence logo and position-frequency matrix of the TATA box. The sequences of the TATA boxes exhibited a certain extent of variation in the PRV genes. The sequence logos were generated by using WebLogo [74]. (B) The amounts of transcripts produced by the various TATA box variants. We found a varying quantity of transcripts produced from the different TATA boxes. Bars represent the average ROIs produced by the given TATA box variants. The highest level of expression is produced by the TATCAGT sequence of the ul10 gene. The expression ratios were calculated from the data generated using the non-amplified library preparation technique. TATA boxes labeled by asterisks are identified by the amplified method. (C) The number of genes using the particular TATA box variants.
(PDF)

S3 Fig Distribution of GC content in the three reading frames. In the PRV genome the third codon positions within the ORFs contain almost exclusively G or C bases, which provides a reliable method for the identification of coding sequences.
*The GC-frame plots of the orf-1 gene cluster exhibit a moderate bias of GC distribution at the third codon position*.*The GC-distribution is not biased at the CTO region*.*The GC-content of noir genes is not higher at any reading frames*.*There is no GC-preference at any of the reading frames in the pto genes*. *The GC-preference of the azure gene in one of its reading frames is the result of its overlap with the us3 gene (there is no GC-preference in the non-overlapping region)*
*Images were generated by Artemis "GC Frame Plot" sequence analysis software*, *release 16*.*0*.*0*.[43].
(PDF)

S4 Fig Log scale representation of the transcriptional landscape of PRV. Transcript abundance is proportional to the number of ROIs.
(PDF)

S1 Table List of the primers used in this study. (XLS)

S2 Table Novel transcripts and transcript isoforms of pseudorabies virus. This table contains novel transcripts and transcript isoforms including mono- and polycistronic transcription units. The already identified transcripts—many with novel transcriptional annotations—are listed in S2 Table.
Abbreviations: TSS: transcription start site; TES: transcription end site
The novel sequences annotated in silico are underlined.
*A*: *Transcription start site—based on the most frequent 5' end**B*: *Transcription end site—based on the most frequent 3' end**C*: *Positions of other promoter elements (GC or CAAT boxes) are indicated if the TATA box was not predicted*. *The positions of these elements are underlined*.*D*: *The uncertain TATA box positions and sequences are labeled by asterisks**E*: *The uncertain PolyA signal positions and sequences are labeled by asterisks*.
(XLS)

S3 Table The list of previously identified genes, many with novel transcriptional annotations. Our study involved the transcriptional annotation of known gene products, which were not annotated with base-pair precision before.
*A*: *In silico prediction of TESs and TATA boxes as in KJ717942*.*1**B*: *Transcription start site—based on the most frequent 5' end**C*: *Transcription end site—based on the most frequent 3' end**D*: *Positions of other promoter elements (GC or CAAT boxes) are indicated if the TATA box was not predicted*. *The positions of these elements are underlined*.*E*: *The uncertain TATA box positions and sequences are labeled by asterisks**F*: *References of the previously experimentally annotated transcripts*.
(XLS)

S4 Table List of genes possessing alternative promoters and poly(A) signals. The uncertain promoter and poly(A) signals are labeled by asterisks. A: Positions of other promoter elements (GC or CAAT boxes) are written if the TATA box was not predicted. The positions of these elements are underlined.
(XLS)

S5 Table Polymorphism at the transcription start and end sites of the abundant transcripts. PacBio analysis revealed that most viral transcripts exhibit a certain extent of variation in both their TSSs and TESs.
(XLS)

S6 Table List of novel putative non-coding genes. This Table contains the 19 newly identified non-coding transcripts of PRV. The lengths, TATA boxes and poly(A) signal sequences of these transcripts are shown. The detected poly(A) tails are also indicated by ‘✓’. A: Positions of other promoter elements (GC or CAAT boxes) are written if the TATA box was not predicted. The positions of these elements are underlined. B: The uncertain poly(A) signals are labeled by asterisks.
(XLS)

S7 Table List of novel mono- and polycistronic RNAs. This Table shows the novel mono-, bi-, tri- and tetracistronic transcripts and their lengths.
(XLS)

S8 Table This table shows the percentages of the antisense-sense transcripts using qPCR (6 h after low and high MOI of PRV infection) data. PK-15 cells were infected with the PRV-Ka strain at different MOIs (0.1 and 10 pfu/cell). Real-Time PCR data were normalized to 28S RNAs.
(DOC)
