# Supplementary figures and images for: Full-Length Isoform Sequencing Reveals Novel Transcripts and Substantial Transcriptional Overlaps in a Herpesvirus
Source: PLoS One. 2016 Sep 29;11(9):e0162868. doi: 10.1371/journal.pone.0162868 (PMC5042381; doi:10.1371/journal.pone.0162868)

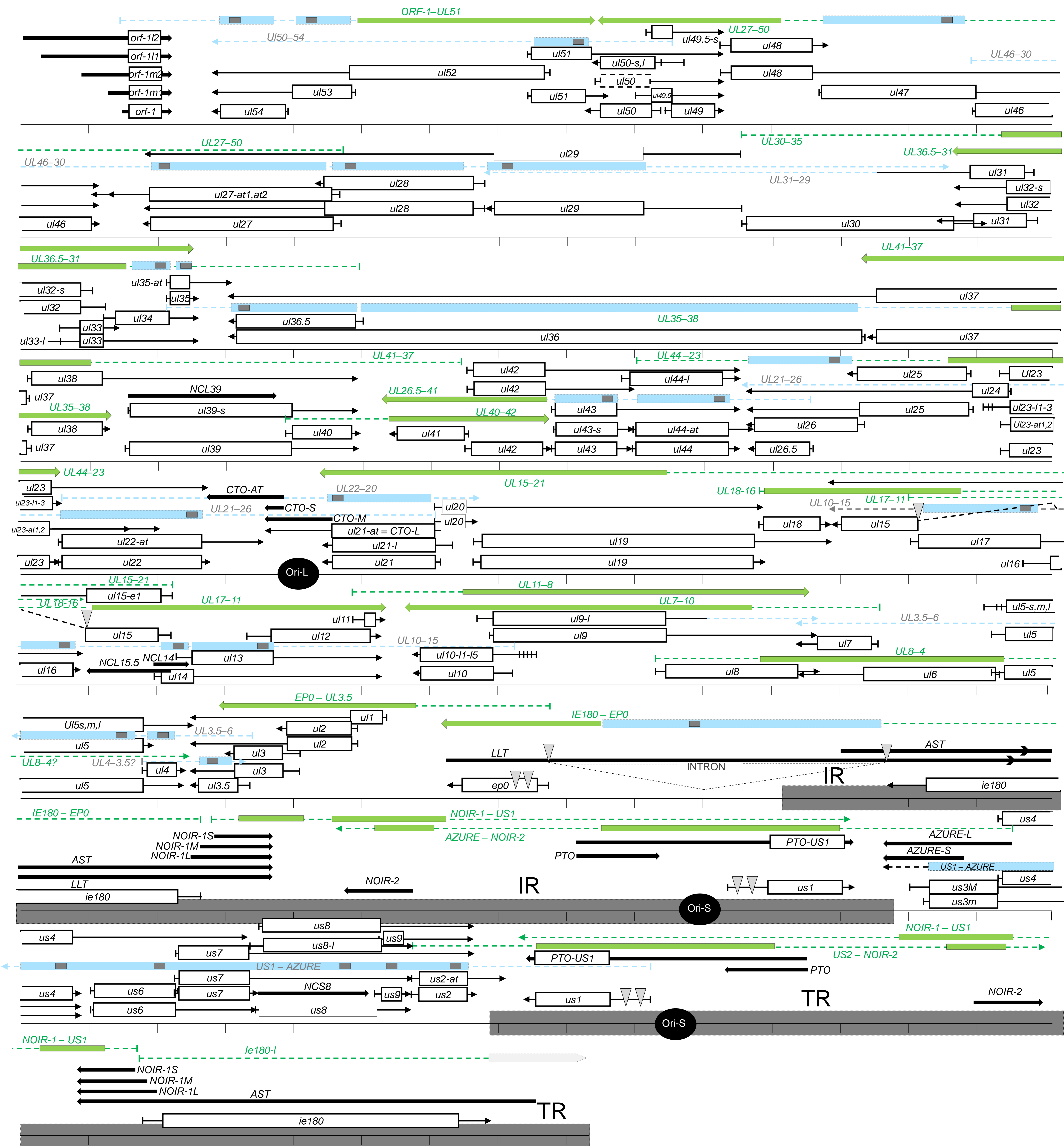

Supplement: S1 Fig — Using PacBio and Illumina RNA sequencing and RT2-PCR, we have detected several very long transcripts with unidentified ends. The transcripts with unknown upstream, or upstream and downstream sequences are depicted as thin green rectangles and dashed arrow-lines in their non-sequenced regions. The green rectangles represent the longest sequence obtained by PacBio sequencing. We assume that the long transcripts are controlled by the next available promoter oriented in the appropriate direction. The 3’ transcript ends, if undetected, were predicted to be located at the next available PAS. However, these assumptions remain to be confirmed experimentally. The names that we selected for these transcripts are therefore considered to be ad hoc (the names contain all of the genes located on a transcript, but in this Figure they are abbreviated by using the names of only the first and last genes). The light-blue rectangles with dashed borders are those parts of the transcripts which we had earlier sequenced (but not published) by the Illumina platform, and the dark-gray rectangles inside them are the genomic loci which were analyzed by RT2-PCR in this study. The protein-coding transcripts are depicted as white rectangles with black borders, while the non-coding RNAs are illustrated as black arrow lines. (PDF) [file pone.0162868.s001.pdf]

A

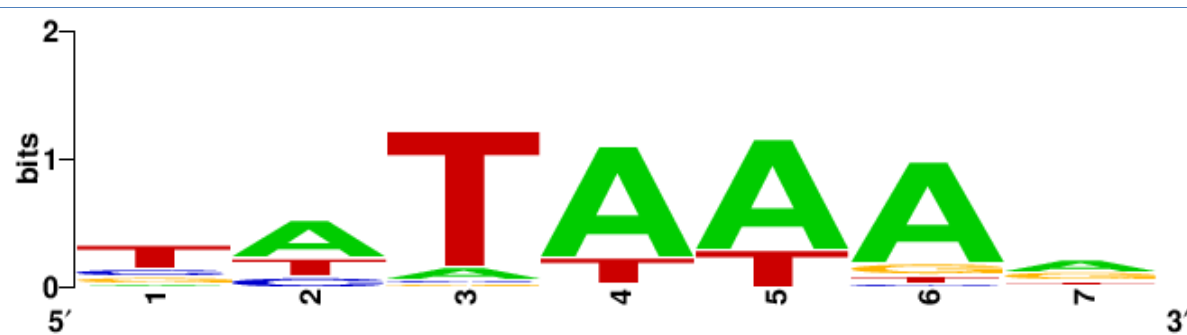

B

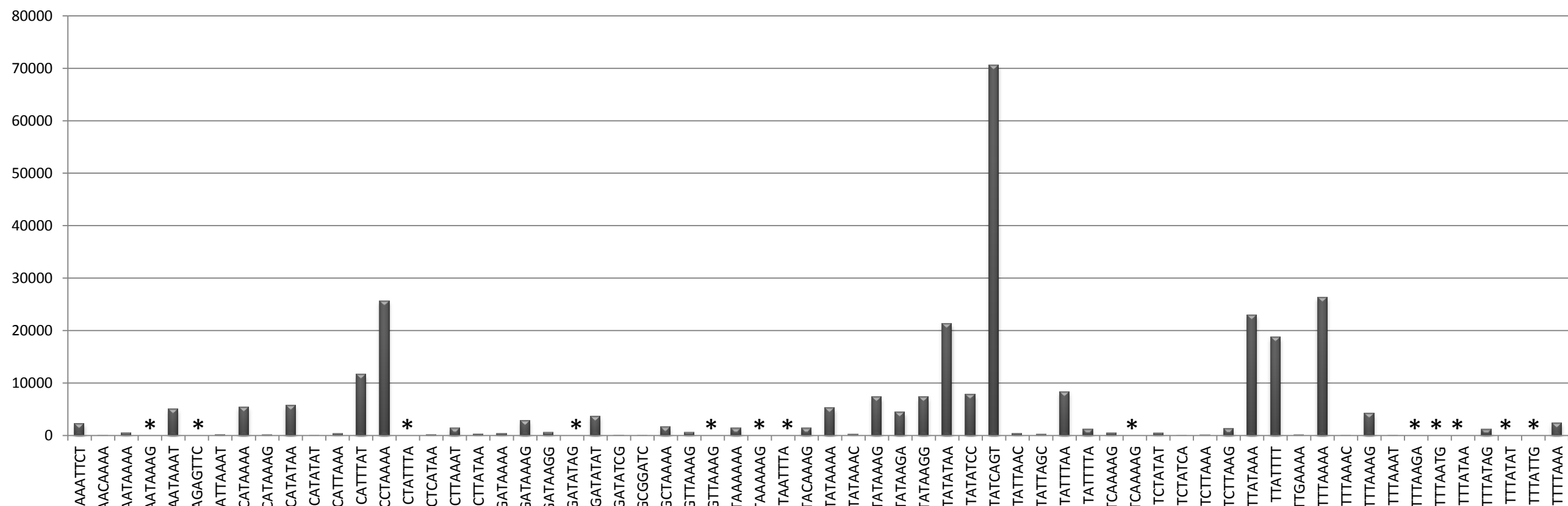

C

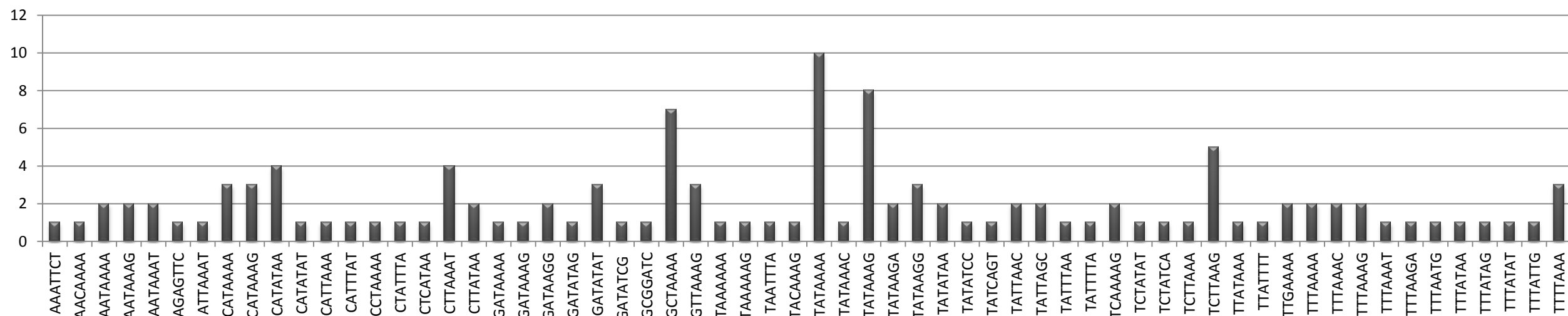

Supplement: S2 Fig — (A) Sequence logo and position-frequency matrix of the TATA box. The sequences of the TATA boxes exhibited a certain extent of variation in the PRV genes. The sequence logos were generated by using WebLogo [74]. (B) The amounts of transcripts produced by the various TATA box variants. We found a varying quantity of transcripts produced from the different TATA boxes. Bars represent the average ROIs produced by the given TATA box variants. The highest level of expression is produced by the TATCAGT sequence of the ul10 gene. The expression ratios were calculated from the data generated using the non-amplified library preparation technique. TATA boxes labeled by asterisks are identified by the amplified method. (C) The number of genes using the particular TATA box variants. (PDF) [file pone.0162868.s002.pdf]

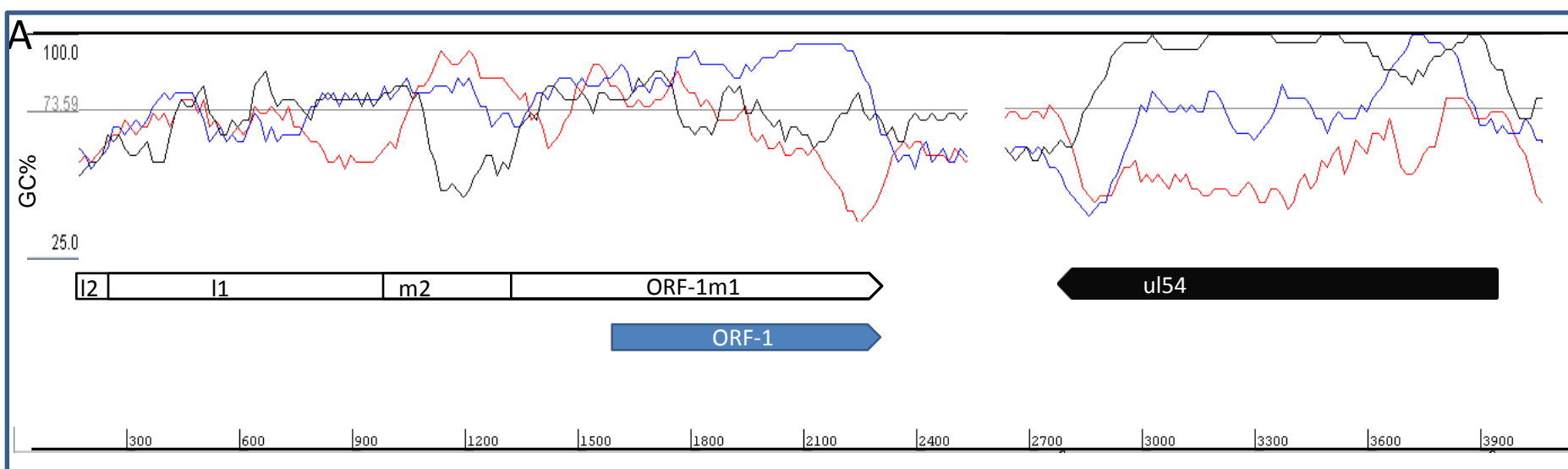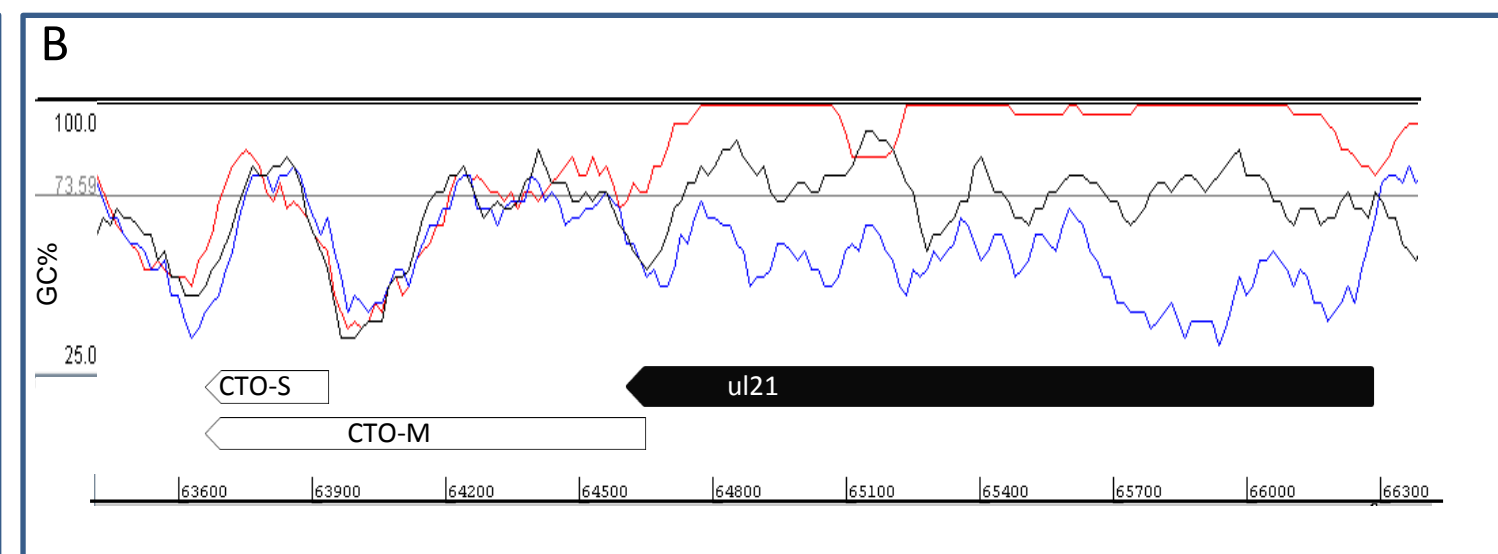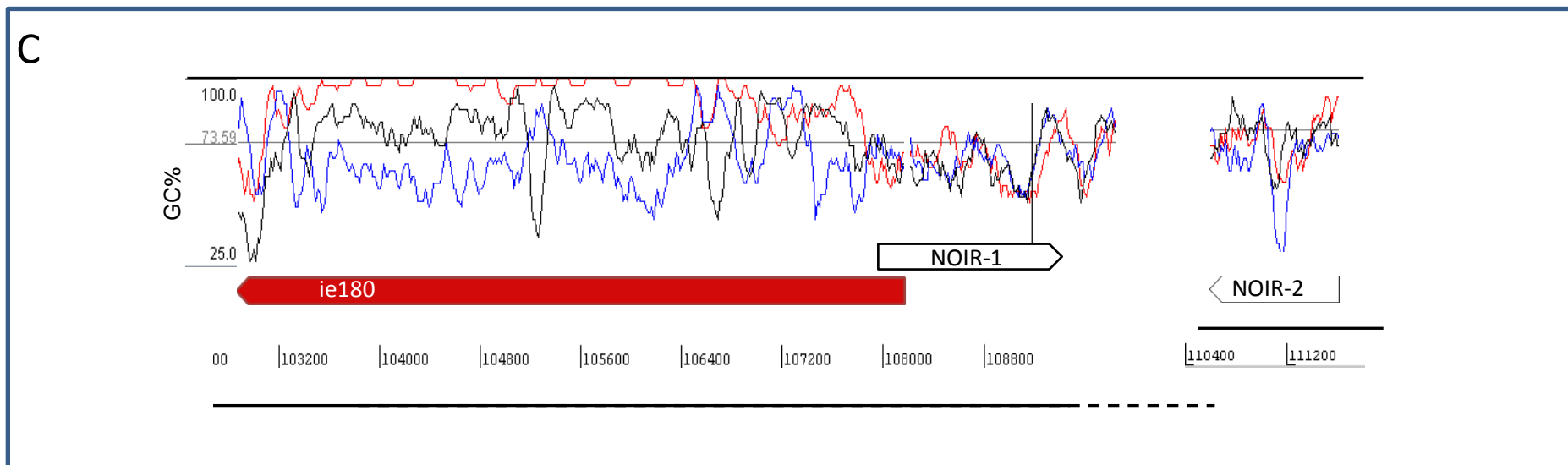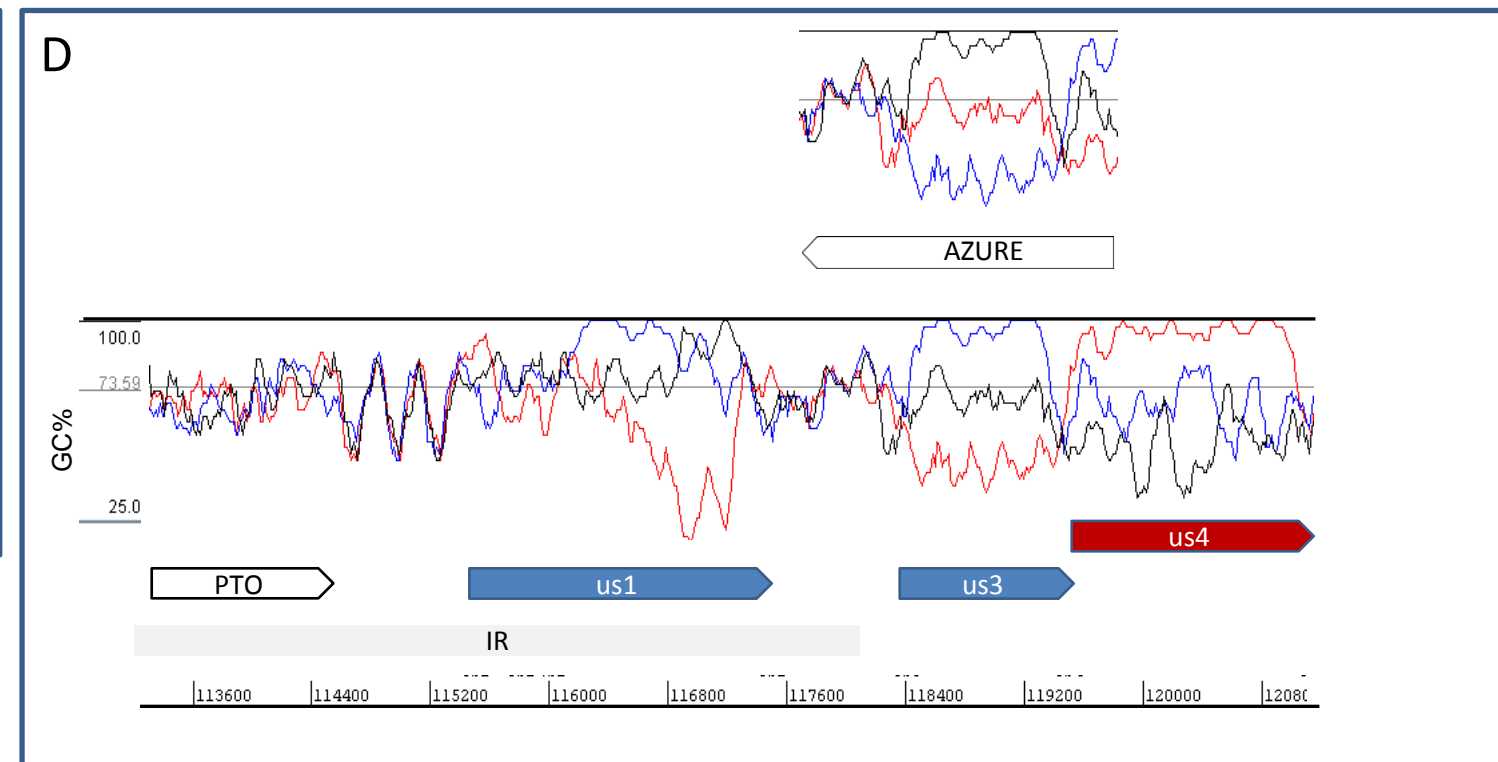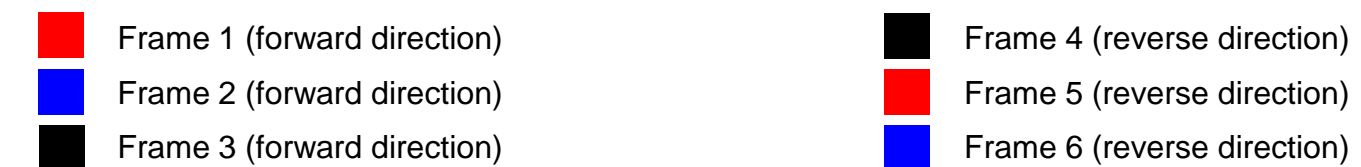

Supplement: S3 Fig — In the PRV genome the third codon positions within the ORFs contain almost exclusively G or C bases, which provides a reliable method for the identification of coding sequences. The GC-frame plots of the orf-1 gene cluster exhibit a moderate bias of GC distribution at the third codon position.The GC-distribution is not biased at the CTO region.The GC-content of noir genes is not higher at any reading frames.There is no GC-preference at any of the reading frames in the pto genes. The GC-preference of the azure gene in one of its reading frames is the result of its overlap with the us3 gene (there is no GC-preference in the non-overlapping region) Images were generated by Artemis "GC Frame Plot" sequence analysis software, release 16.0.0.[43]. (PDF) [file pone.0162868.s003.pdf]

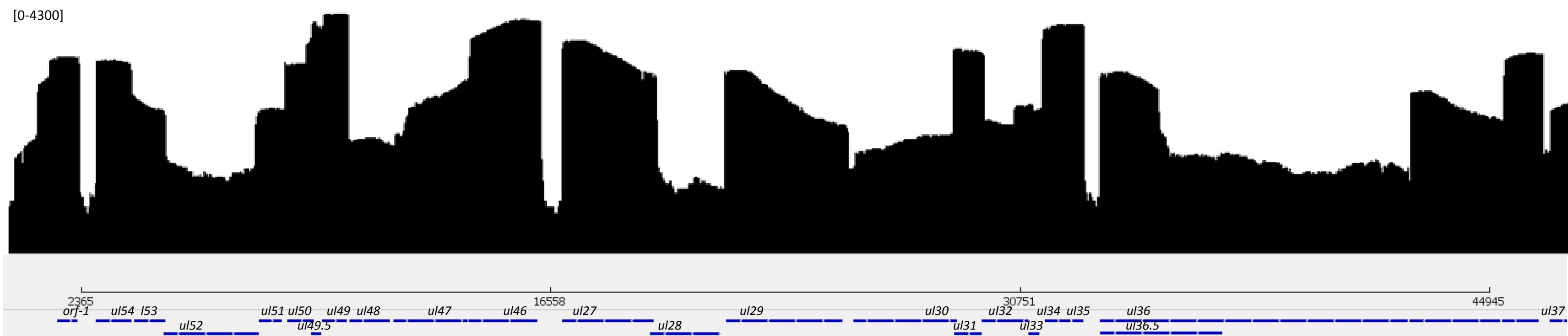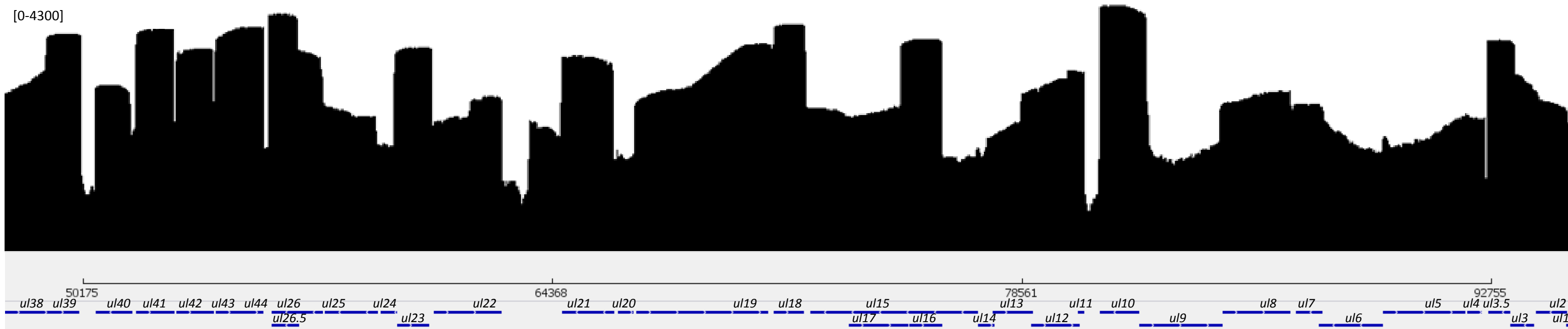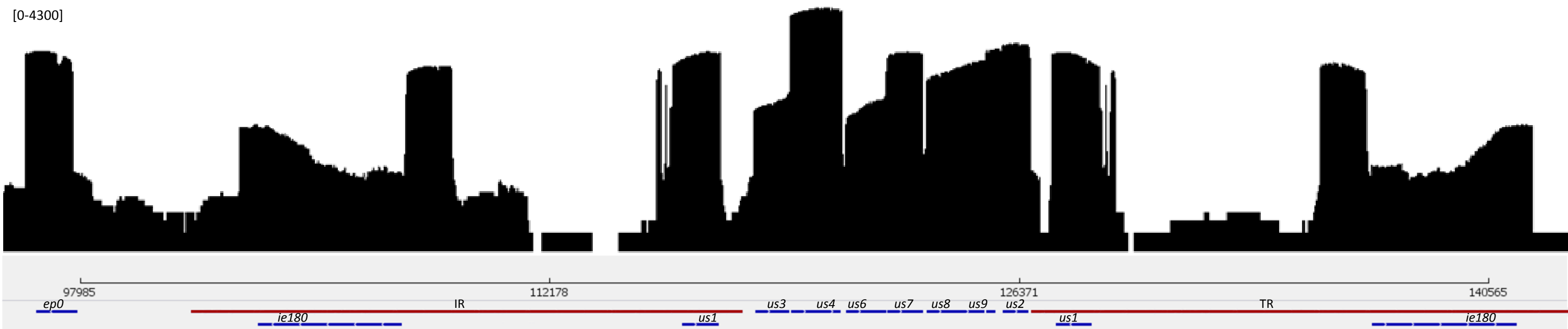

Supplement: S4 Fig — Transcript abundance is proportional to the number of ROIs. (PDF) [file pone.0162868.s004.pdf]
