# Supplementary material for: Full-Length Isoform Sequencing Reveals Novel Transcripts and Substantial Transcriptional Overlaps in a Herpesvirus
Source: PLoS One. 2016 Sep 29;11(9):e0162868. doi: 10.1371/journal.pone.0162868 (PMC5042381; doi:10.1371/journal.pone.0162868)
Supplement: S8 Table — PK-15 cells were infected with the PRV-Ka strain at different MOIs (0.1 and 10 pfu/cell). Real-Time PCR data were normalized to 28S RNAs. (DOC) [file pone.0162868.s012.doc]

|  | **Gene** | **Ratio (asRNA/mRNA) %** | |
| --- | --- | --- | --- |
|  |  | **qPCR**  **10 PFU/cell** | **qPCR**  **0.1 PFU/cell** |
|  | ***ul54*** | 14.90 | 9.38 |
|  | ***ul53*** | 27.92 | 9.26 |
|  | ***ul52*** | 107.28 | 50.25 |
|  | ***ul51*** | 178.64 | 9.16 |
|  | ***ul50*** | 26.15 | 22.17 |
|  | ***ul49.5*** | 3.58 | ND |
|  | ***ul49*** | ND | 4.03 |
|  | ***ul48*** | 5.92 | ND |
|  | ***ul47*** | ND | ND |
|  | ***ul46*** | ND | 3.48 |
|  | ***ul27*** | ND | 4.70 |
|  | ***ul28*** | ND | 135.62 |
|  | ***ul29*** | 3.27 | 7.45 |
|  | ***ul30*** | 149.72 | 77.23 |
|  | ***ul31*** | 7.54 | ND |
|  | ***ul32*** | ND | 47.95 |
|  | ***ul33*** | 8.09 | 14.80 |
|  | ***ul34*** | ND | 1.38 |
|  | ***ul35*** | ND | 1.99 |
|  | ***ul36.5*** | 21.16 | 4.01 |
|  | ***ul36*** | 0.002 | ND |
|  | ***ul37*** | 10.48 | 16.44 |
|  | ***ul38*** | 32.27 | 4.01 |
|  | ***ul39*** | 14.06 | 4.91 |
|  | ***ul40*** | 18.23 | 3.08 |
|  | ***ul41*** | 98.05 | 11.97 |
|  | ***ul42*** | 3.86 | 5.59 |
|  | ***ul43*** | 18.11 | 21.59 |
|  | ***ul44*** | 6.21 | 29.73 |
|  | ***ul26.5*** | ND | 2.09 |
|  | ***ul26*** | ND | 6.01 |
|  | ***ul25*** | ND | 7.45 |
|  | ***ul24*** | 10.02 | 33.14 |
|  | ***ul23*** | 1.08 | ND |
|  | ***ul22*** | 3.29 | 0.89 |
|  | ***cto-s*** | 3.36 | ND |
|  | ***cto-m*** | ND | 13.79 |
|  | ***cto-l*** | ND | 42.40 |
|  | ***ul21*** | 9.93 | 9.81 |
|  | ***ul20*** | 9.62 | 2.94 |
|  | ***ul19*** | 1.64 | 1.24 |
|  | ***ul18*** | ND | 3.71 |
|  | ***ul17*** | 21.04 | 22.05 |
|  | ***ul16*** | ND | ND |
|  | ***ul15.5*** | 555.56 | ND |
|  | ***ul15 (e2)*** | 44.19 | 18.37 |
|  | ***ul14*** | 26.36 | 16.09 |
|  | ***ul13*** | ND | 14.49 |
|  | ***ul12*** | ND | 7.19 |
|  | ***ul11*** | ND | 15.51 |
|  | ***ul10*** | 0.60 | 0.88 |
|  | ***ul9*** | 42.77 | 24.87 |
|  | ***ul8*** | ND | 17.14 |
|  | ***ul7*** | ND | ND |
|  | ***ul6*** | 93.86 | 71.64 |
|  | ***ul5*** | 38.12 | 26.32 |
|  | ***ul4*** | ND | 39.36 |
|  | ***ul3.5*** | ND | 55.70 |
|  | ***ul3*** | ND | 3.74 |
|  | ***ul2*** | ND | 0.20 |
|  | ***ul1*** | 14.65 | 3.39 |
|  | ***ep0*** | 87.45 | 22.94 |
|  | ***ep0-ie180*** | 90.09 | ND |
|  | ***ie180*** | 9.22 | 12.26 |
|  | ***noir-1*** | 58.14 | ND |
|  | ***noir-2*** | 833.33 | ND |
|  | ***pto*** | 96.15 | ND |
|  | ***us1*** | 97.27 | 5.30 |
|  | ***azure*** | 83.86 | ND |
|  | ***us3*** | 42.36 | 35.28 |
|  | ***us4*** | ND | 8.02 |
|  | ***us6*** | 6.67 | 22.30 |
|  | ***us7*** | ND | 13.59 |
|  | ***us8*** | ND | 10.81 |
|  | ***us9*** | 1.71 | 2.44 |
|  | ***us2*** | ND | 3.70 |
